# ﻿*Habralictus* and *Lasioglossum* of Saint Lucia and Saint Vincent and the Grenadines, Lesser Antilles (Hymenoptera, Apoidea, Halictidae)

**DOI:** 10.3897/zookeys.1089.72645

**Published:** 2022-03-18

**Authors:** Jason Gibbs, Amber Bass, Katherine Morgan

**Affiliations:** 1 Department of Entomology, University of Manitoba, 12 Dafoe Road, Winnipeg, Manitoba, Canada University of Manitoba Winnipeg Canada; 2 Current address: Agriculture and Agri-Food Canada, Canadian National Collection of Insects, 960 Carling Avenue, Ottawa, Ontario, Canada Agriculture and Agri-Food Canada, Canadian National Collection of Insects Ottawa Canada

**Keywords:** Anthophila, Caribbean, Halictinae, new species, sweat bees, taxonomy

## Abstract

The new species and the first halictid bees documented from Saint Lucia *Habralictusreinae*, Lasioglossum (Dialictus) luciae, and L. (Habralictellus) delphiae are described. A fourth species, L. (D.) dominicense, is tentatively recorded from the island. The species are illustrated and compared to similar ones from the Lesser Antilles. *Lasioglossum* and *Habralictus* from neighbouring Saint Vincent and the Grenadines are reviewed and a key to *Lasioglossum* provided, including the description of another new species, *L. (Dialictus) gemmeum. Trigonanigrocyanea* Ashmead and *Dufoureasubcyanea* Ashmead are synonymised under *Lasioglossumcyaneum* (Ashmead). Notes on the obscure Lasioglossum (Dialictus) minutum (Fabricius) are provided. A new name, Lasioglossum (Homalictus) minuens, is provided for a secondary homonym *Homalictusminutus* Pauly. The potential for additional species richness in Saint Lucia and the Lesser Antilles is briefly discussed.

## ﻿Introduction

The bees of the Caribbean Islands have received sporadic attention from melittologists. Despite the idyllic landscape of these islands, the lack of species richness may have dissuaded many researchers from visiting. However, specimens accumulated in museum collections have allowed for some recent studies on the regional bee fauna. Bees on the major islands in the Greater Antilles, Cuba, Hispaniola, and Puerto Rico, have been documented relatively well (Baker 1906; Alayo 1973, 1976; Eickwort 1988; Genaro 2001a, b, 2006, 2007, 2008, 2016; Engel 2006a; Genaro and Franz 2008; Engel and Prado 2014; Gibbs 2018). However, recent discoveries of new species (Genaro 2016, 2021; Gibbs 2018) suggest that more diversity may be present throughout the Caribbean Islands.

The numerous small islands that make up the Lesser Antilles are generally less well-known for bees. Recent studies in the French West Indies, Guadeloupe and Martinique, have documented bees in the Apidae and Megachilidae (Meurgey 2014, 2016; Meurgey and Dumbardon-Martial 2015). The species list for the Halictidae remains at zero, although halictid bees do occur on these islands (Gibbs 2016; Meurgey 2016). Dominica, which lies immediately between Guadeloupe and Martinique, has recently had its halictid fauna revised with 11 species in five genera documented (Gibbs 2016). Saint-Vincent and the Grenadines has 16 halictid species known (Ashmead 1900; Cockerell 1910; Moure 2007; Ascher and Pickering 2021). It seems reasonable to conclude that islands to the south of Dominica, i.e., Martinique and St. Lucia, should have a comparable fauna of halictid bees.

Saint Lucia is an island of similar size (617 km^2^) to Dominica (750 km^2^), which lies between Martinique and Saint Vincent and the Grenadines (SVG). Saint Lucia currently has a rather depauperate faunal list of six bee species (Moure et al. 2007a; Raw 2007; Ascher and Pickering 2021), including the apids *Apismellifera* L. 1758, *Centrisdecolorata* Lepeletier 1841, *Centrisversicolor* (Fabricius 1775), and *Mesopliaazurea* (Lepeletier and Audinet-Serville 1825) and the megachilids *Megachilederelictula*Cockerell 1937 and *M.lanata* (Fabricius 1775). *Apismellifera* and both *Megachile* are non-native. The first known halictid bees from the island are documented herein. In comparing these new species to bees from neighbouring islands (Ashmead 1900; Smith-Pardo 2009; Gibbs 2012, 2016), we also clarify the taxonomy of some *Lasioglossum* from SVG. Ashmead (1900) first documented and described the bee fauna from SVG. There has since been little additional taxonomic work on *Lasioglossum* on the island (but see Moure 2007). We describe a new species from SVG, propose two synonymies, and remove one additional name from the fauna.

## ﻿Materials and methods

Many specimens from various collections have been examined for taxonomic studies of Caribbean Halictidae, particularly *Lasioglossum* but also *Habralictus* (Gibbs 2012, 2016, 2018). Saint Lucia material was found at the American Museum of Natural History (**AMNH**), Florida State Collection of Arthropods (**FSCA**), Montana Entomology Collection, Montana State University (**MTEC**), and the National Museum of Natural History, Smithsonian Institution (**USNM**). These species are described to formally document the family Halictidae from the island. Material from Saint Vincent and the Grenadines was examined from FSCA, National Museum of Natural History, Smithsonian InstitutionUSNM, Natural History Museum (**NHMUK**), University of Kansas Biodiversity Institute and Natural History Museum (**SEMC**), Packer Collection York University, and J.B. Wallis / R.E. Roughley Museum of Entomology (**WRME**). The Packer Collection specimens were returned without full data recorded, but a subset was deposited at WRME.

Species descriptions follow the format of recent papers on Caribbean *Lasioglossum* (Gibbs 2016, 2018), with some modifications based on Gardner and Gibbs (2020). Terminology for structures follows Michener (2007) with modifications based on Engel (2001a) for wing venation and Gibbs (2010a) for the propodeum. Surface sculpturing follows that of Harris (1979). The term ‘granular’ is used for the surface sculpturing of *Habralictus* following (Michener 1979; Smith-Pardo 2009; Gibbs 2012), although at high magnifications (150×) it seems this granular effect is due to the surface being microreticulate dorsally, i.e., composed of a close network of raised lines, whereas on the pleura the granular sculpturing is more imbricate.

Measurements for head length, head width, clypeal length, lower interocular distance (**LOD**), and upper interocular distance (**UOD**) follow Michener (2007). All measurements were taken using an ocular micrometer in an Olympus SZX16 microscope at 50–63× magnification or 115× for antennae. Body length was measured by adding the length from the base of antenna to the apex of the propodeum with the length of the metasoma. Face length was measured from the clypeal apex to the lower margin of the median ocellus. Antennal measurements were taken on the shortest side of flagellomere two. Intertegular distance (**ITD**) was the smallest distance between the tegulae in dorsal view. Mesoscutal length was the medial length taken in the same orientation as the ITD. Mesoscutellar, metanotal, and propodeal lengths were measured such that the propodeal posterior surface was parallel to the line of sight. Wing length was measured from the proximal end of the basal vein (**M**) to the apex of the marginal cell. Puncture density is measured in terms of relative spacing given as the length of interspaces (IS) between punctures relative to the puncture diameter (**PD**). Setal length is given in terms of mid ocellus diameters (**MOD**). Metasomal terga and sterna are abbreviated with **T** and **S**, respectively, followed by the appropriate number counting from the proximal segment. Similarly, flagellomeres are abbreviated with **F** followed by the appropriate number.

## ﻿Systematics

### ﻿Genus Habralictus

Taxon classificationAnimaliaHymenopteraHalictidae

Moure, 1941

Habralictus Moure 1941: 59. Type species: Habralictusflavopictus Moure 1941, by original designation
Zikaniella Moure 1941: 57. Type species: Zikaniellacrassiceps Moure 1941, by original designation

### ﻿ Habralictus reinae sp. nov.

Taxon classificationAnimaliaHymenopteraHalictidae

http://zoobank.org/F1285ABB-2BB0-49FC-A551-7CDD6ECA0D33

Figs 1 , 2 , 3A

#### Holotype.

**Saint Lucia** • **Micoud District** • Quilesse Forest Reserve, Laporte, 13.8404, -60.9741, 272 m, 5–7.V.2009, leg. I.A. Foley and R.C. Winton, UV light trap (♂ MTEC, to be deposited in the USNM).

#### Paratypes.

**Saint Lucia** • **Castries District** • Barre de l’Isle, 13.93682, -60.95936, 340 m, 25­–28.VI.2009, leg. E.A. Ivie, UV light trap (1 ♀ MTEC) • Barre de l’Isle, 13.93682, -60.95936, 340 m, 8–14.VII.2009, leg. C.A. Maier and M. Gimmel, UV light trap (1 ♂ MTEC) • Barre de l’Isle, 13.9342, -60.9586, 340 m, 22­–29.V.2009, leg. R.C. Winton, Malaise trap (1 ♀ WRME) • Barre de l’Isle, 13.9342, -60.9586, 340 m, 27.VI­–3.VII.2009, leg. C.A. Maier and M. Gimmel, UV light trap (1 ♂ MTEC) • **Micoud District** • Quilesse Forest Reserve, Laporte, 13.8404, -60.9741, 272 m, 5–7.V.2009, leg. I.A. Foley and R.C. Winton, UV light trap (3 ♂ MTEC, 2 ♂ WRME).

**Figure 1.**
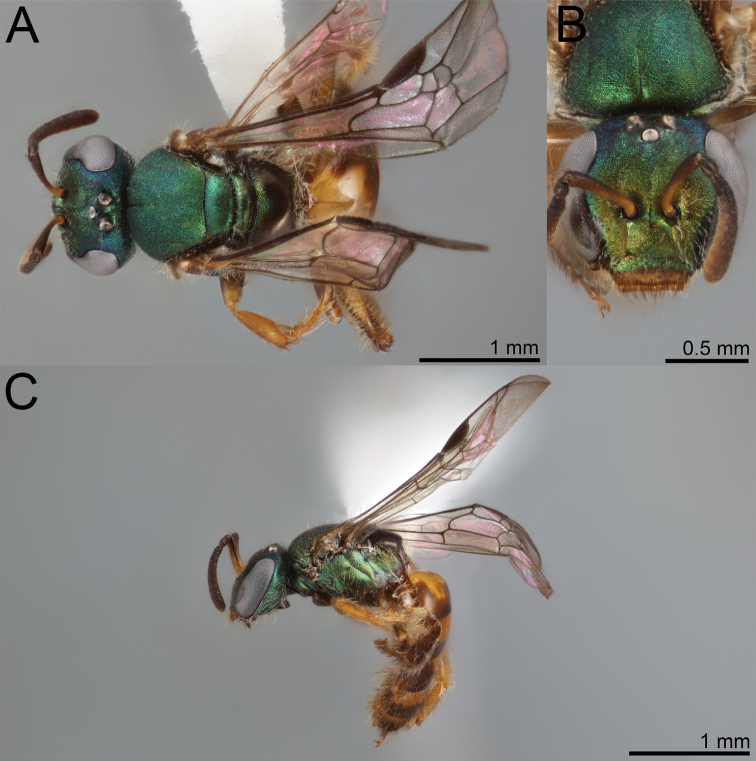
*Habralictusreinae* sp. nov., paratype female **A** dorsal habitus **B** head, frontal view **C** lateral habitus.

#### Diagnosis.

Males of *H.reinae* can be distinguished from other *Habralictus* in the Lesser Antilles by the combination of head narrow (length/width ratio = 1.0–1.07) (length/width ratio = 0.84–0.85 in *H.antillarus*), clypeus with distal maculation 1/3–1/2 longitudinal length (< 1/5 length in *H.antillarus*), supraclypeal and lower paraocular areas polished due to lack of microsculpture (distinctly imbricate in *H.gonzalezi*), mesoscutal punctation indistinct (fine but distinct in *H.claviventris* and *H.insularis*); mesepisternum polished with only weak microsculpture, sparse punctures distinct (dull, indistinctly punctate in *H.gonzalezi*).

**Figure 2.**
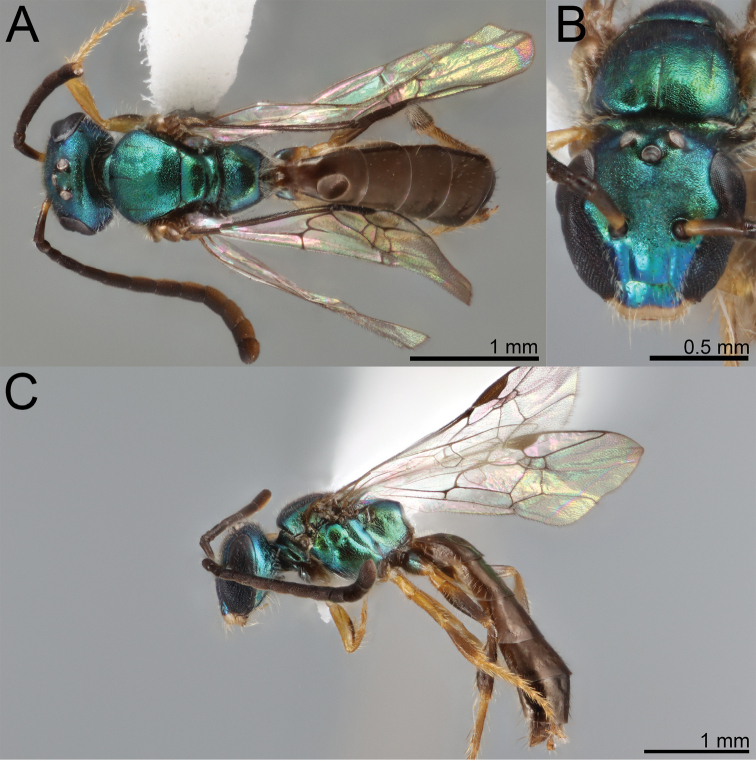
*Habralictusreinae* sp. nov., paratype male **A** dorsal habitus **B** head, frontal view **C** lateral habitus.

**Figure 3.**
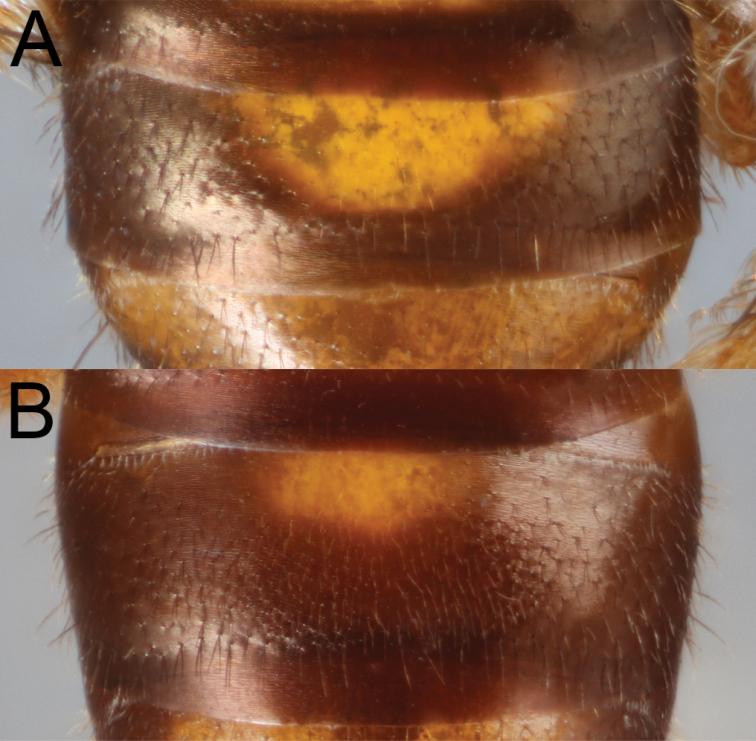
Metasomal tergum 3 of **A***Habralictusreinae* sp. nov. **B***Habralictusgonzalezi* Gibbs, 2012 to illustrate differences in setose puncture density.

Females of *H.reinae* can be recognised by the combination of head wide (length/width ratio = 9.0) (length/width ratio = 0.92–0.97 in *H.gonzalezi*), clypeal punctures not distinct (distinctly punctate in *H.insularis*), clypeal maculation ½ length of clypeus (1/3 in *H.antillarus*) and T3 sparsely punctate (Fig. 3A) as in T4 (more densely punctate in *H.gonzalezi*; Fig. 3B). The female of *H.claviventris* is unknown.

#### Description.

**Female** (*n* = 2). Length 4.3–4.5 mm; head length 1.1 mm; head width 1.2 mm; intertegular distance 0.86–0.89 mm; wing length 1.6–1.8 mm.

***Colouration*.** Head and mesosoma bright metallic blue-green with golden and bronze reflections. Clypeal apex pale brownish yellow, base bronze. Labrum reddish brown. Mandible yellow with black base and red apex. Scape yellow ventrally, brown dorsally. Flagellum dark brown, F2-F11 orange-brown ventrally. Pronotal lobe brown. Tegula yellowish brown. Wing membrane faintly dusky, veins brown to dark brown. Legs with varying brown and yellow, brown primarily on coxa, femur and meso- and metatibiae, yellow on trochanters, profemur apex, protibia and protarsi, dorsal or anterior surface of mesotibia, and posterior surface of metatibia and variably on posterior surface of metafemur, Metasoma brown and yellow-orange, yellow-orange on base of terga and on sterna, apical terga brown.

***Pubescence*.** Body with sparse pilosity, dull white to faintly yellowish, dark setae on meso- and metatibia, and scattered on T4–T6. Tomentum on pronotal dorsolateral angles and lobe. Mesoscutal pilosity sparse erect. Wing setae dark. T1 without appressed fan. Terga with sparse setae, absent on apical impressed areas.

***Surface sculpture*.** Clypeal punctures indistinct, sparse (IS = 2–4 PD), denser along apical margin (IS = 1–2 PD), interspaces granular. Face granular with indistinct punctation. Gena imbricate. Tegula punctures obscure. Mesoscutum and mesoscutellum granular with indistinct punctation. Metapostnotum granular, microreticulate basally becoming imbricate toward margin. Mesopleuron granular (imbricate). Propodeal lateral face imbricate, sparsely punctate; posterior face imbricate, sparsely punctate. Metasomal terga finely coriarious. sparse setose punctures (IS = 3–6 PD) along premarginal line of T2-T4 and disc of T3-T5, apical impressed areas impunctate.

***Structure*.** Face length/width ratio 0.78 (± 0.01 SD). UOD/LOD ratio 1.06 (± 0.11 SD). Clypeus projecting ~75% below suborbital tangent; apicolateral denticles rounded knobs. Supraclypeal area length/width ratio 0.97 (± 0.11 SD). Hypostomal carinae parallel. Pronotal angle obtuse. Mesoscutum length/width ratio 0.91 (± 0.06 SD); mesoscutum/mesoscutellum length ratio 2.93(± 0.33 SD); mesoscutellum/metanotum length ratio 1.73 (± 0.13 SD); metanotum/metapostnotum length ratio 0.5 (± 0.03 SD). Lateral propodeal carinae reaching dorsolateral slope; oblique carina absent. Tegula shape ovoid. Forewing with 3 submarginal cells. Distal hamuli arranged 2-1-2. Inner metatibial spur pectinate, with 3 branches not including apex of rachis, proximal branch much longer than width of rachis. Metasoma ovoid, dorsoventrally flattened, apical impressed area medially ~ 1/2 longitudinal length of basal area.

**Male** (*n* = 3). Length 4.0–4.3 mm; head length 0.98–1.06 mm; head width 0.94–1.05 mm; intertegular distance 0.65–0.71 mm. Similar to female with usual sex associated modifications.

***Colouration*.** Head and mesosoma iridescent blue-green. Clypeus pale yellow on apical third. Labrum pale yellow. Mandible pale yellow, orange apically. Flagellum brown, F3-F11 yellowish brown ventrally. Pronotal lobe brown. Tegula translucent amber. Wing membrane faintly dusky, veins dark brown. Pro- and mesoleg yellow, except coxa dark with weak metallic reflections, femora ventrally and mesotibia infused with brown. Meta leg brown, except coxa metallic, and trochanter, apices and bases of femur and tibia, and tarsi yellowish brown. Metasoma brown, apical impressed areas reddish brown.

***Pubescence*.** Body pilosity sparse, dull white to faintly yellowish. Gena with long setae (2–2.5 OD). Pronotal lobe with tomentum on posterior margin. Mesoscutal setae sparse, short (0.5 OD). Metasomal terga largely bare; sternal setae sparse (1–1.5 OD), moderately plumose, sparse, erect. Wing setae dark, short, sparse.

***Surface sculpture*.** Clypeal punctures sparse (IS = 1–2 PD), interspaces shiny, weakly imbricate. Supraclypeal punctures sparse (IS = 1–3 PD), interspaces shiny, weakly imbricate. Lower paraocular punctures sparse (IS = 1–3 PD), interspaces shiny, weakly imbricate. Frons and upper paraocular area granular. Gena punctulate-polished; postgena shiny, weakly imbricate. Tegula mostly impunctate. Mesoscutal punctation indistinct, interspaces granular. Mesoscutellar punctation moderately sparse (IS = 1–1.5 PD), interspaces strongly imbricate. Metanotum punctate, interspaces imbricate. Metapostnotum finely reticulate-granular. Pre-episternum imbricate. Hypoepimeral area punctate (IS = 1–1.5 PD), interspaces shiny imbricate. mesepisternum finely punctate (IS = 1–4 PD), interspaces shiny imbricate. Metepisternum imbricate. Propodeum imbricate. Metasoma sparsely punctate (IS = 5–10 PD), apical impressed areas impunctate, interspaces coriarious.

***Structure*.** Face length/width ratio 0.86 (± 0.05 SD). F1: pedicel length ratio 1.1. F2:F1 length ratio 2.5. Gena narrower than eye. Hypostomal carinae parallel. Pronotal angle obtuse. Mesoscutum length/width ratio 0.97 (± 0.03 SD); mesoscutum/mesoscutellum length ratio 2.96 (± 0.05 SD); mesoscutellum/metanotum length ratio 1.92 (± 0.14 SD); metanotum/metapostnotum length ratio 0.5 (± 0.03 SD). Lateral propodeal carina nearly reaching dorsal margin; oblique carina absent. Tegula ovoid. Forewing with 3 submarginal cells. Metatibial spurs ciliate. Metasoma slender, clavate, widest at T4.

#### Etymology.

This brilliant, shining bee is appropriately named for Reina Rybuck, a curious and inquisitive girl who loved insects. Her light shone bright but too briefly. She is remembered with love and affection by those who knew her.

#### Notes.

Of the five *Habralictus* species known from the Lesser Antilles, all seem to be limited to higher elevations (272–762 m) on the islands (Ashmead 1900; Smith-Pardo 2009; Gibbs 2012, 2016). *Habralictusreinae* was taken from protected canopy forests that are particularly wet. It is notable for future collection efforts that this species was predominantly collected from UV light traps, despite more frequent use of Malaise traps by collectors and daytime net collecting (M. Ivie, in litt.).

### ﻿ Habralictus claviventris

Taxon classificationAnimaliaHymenopteraHalictidae

(Ashmead 1900)

Fig. 4

Augochlora claviventris (1900: 217). Saint Vincent – windward side. 1500 feet. Holotype male by monotypy (NHMUK: BMNH 17.a.1037).
Augochlora claviventris ­: Ashmead (1900: 304) checklist; Friese (1909: 38) catalogue; Cockerell (1910: 489, 494) checklist, taxonomic notes).
Habralictus claviventris : Michener (1979: 181) new combination, checklist; Moure and Hurd (1987: 174) catalogue; Moure (2007: 837) catalogue; Smith-Pardo (2009: 53) taxonomic notes; Gibbs (2012: 3,9) taxonomic notes, key.

#### Notes.

Currently only known from the holotype male.

**Figure 4.**
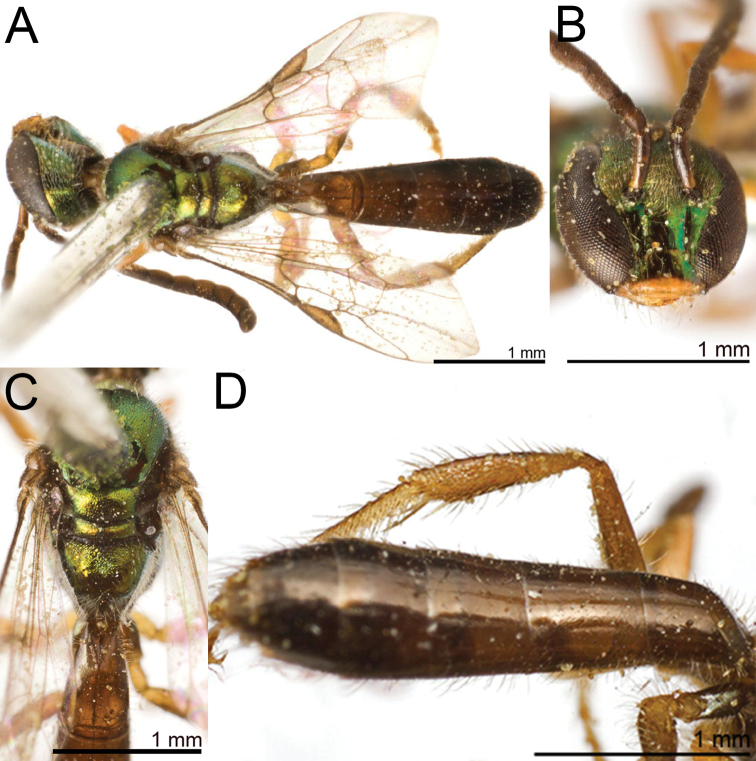
*Habralictusclaviventris* Ashmead, 1900, holotype male **A** dorsal habitus **B** face, oblique frontal view **C** dorsal view of metasoma and tergum 1 **D** dorsolateral view of metasoma. Images courtesy of the Trustees of the Natural History Museum, London (https://creativecommons.org/licenses/by/4.0/).

##### Genus *Lasioglossum* Curtis, 1833

### Subgenus﻿ Dialictus

Taxon classificationAnimaliaHymenopteraHalictidae

Robertson, 1902

Paralictus Robertson 1901: 229. Type species: Halictuscephalicus Robertson, 1892, by original designation
Dialictus Robertson, 1902a: 48. Type species: Halictusanomalus Robertson, 1892, by original designation and monotypy
Chloralictus Robertson, 1902c: 248. Type species: Halictuscressonii Robertson, 1890, by original designation
Halictus (Gastrolictus) Ducke, 1902: 102. Type species: Halictusosmioides Ducke, 1902, by monotypy
Halictomorpha Schrottky, 1911: 81. Type species: Halictomorphaphaedra Schrottky, 1911, by original designation
Rhynchalictus Moure, 1947: 5. Type species: Rhynchalictusrostratus Moure, 1947, by original designation
Halictus (Smeathhalictus) Warncke 1975: 88. Type species: Melittasmeathmanella Kirby, 1802, by original designation
Lasioglossum (Afrodialictus) Pauly 1984: 142. Type species: Halictusbellulus Vachal, 1909, by original designation
Gnathalictus Moure 2001: 493. Type species: Gnathalictuscapitatus Moure, 2001, by original designation
Evylaeus (Viridihalictus) Pesenko 2007: 25. Type species: Halictusviridis Brullé, 1840, by original designation
Evylaeus (Glauchalictus) Pesenko, 2007: 26. Type species: Halictusproblematicus Blüthgen, 1823, by original designation
Evylaeus (Virenshalictus) Pesenko, 2007: 26. Type species: Hylaeusvirens Erichson, 1835, by original designation
Evylaeus (Loethalictus) Pesenko, 2007: 26. Type species: Halictusloetus Brullé, 1840, by original designation
Evylaeus (Aerathalictus) Pesenko, 2007: 27. Type species: Melittaaerata Kirby, 1802, by original designation

### ﻿ Lasioglossum (Dialictus) luciae sp. nov.

Taxon classificationAnimaliaHymenopteraHalictidae

http://zoobank.org/BF175658-2800-4F30-94FE-6B5743544478

Figs 5 , 6

#### Holotype.

**Saint Lucia** • **Castries District** • Piton Flore, Station no. 26, 10.I.1975, leg. J. Hance & G. Whitmyre (♂ FSCA).

#### Paratypes.

**Saint Lucia** • **Castries District** • Castries, 0–210 m, VIII.1976, N.L.H. Krauss (2 ♀ AMNH); Castries, 10–22.IX.1919, leg. J.C. Bradley (2 ♂ USNM) • **Micoud District** • Escap Community, Fond Bay at beach, 13.8316, -60.893, 1 m, 8.V.2009, leg. C.M. Delphia and J.B. Runyon (1 ♀ MTEC).

#### Diagnosis.

*Lasioglossumluciae* is one of only two L. (Dialictus) known from St. Lucia. It can be distinguished from L. (D.) dominicense by the larger size and longer head. It resembles *L.kilpatrickae* Gibbs, 2016 from Dominica and both *L.plumbeum* (Ashmead, 1900) and *L.sanctivincenti* (Ashmead, 1900) from Saint Vincent and the Grenadines.

**Figure 5.**
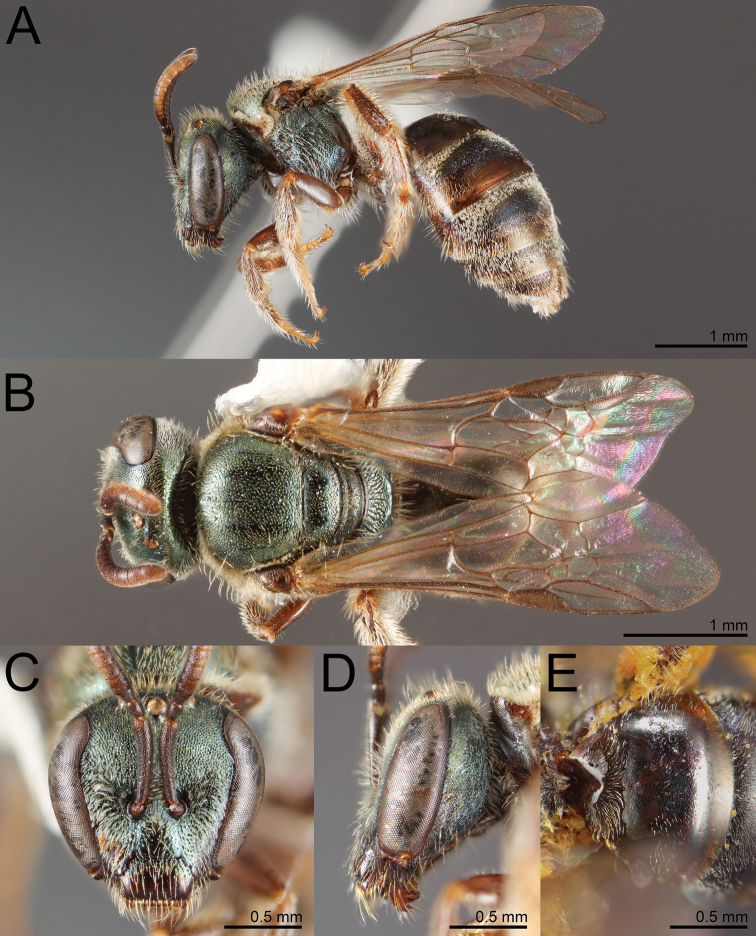
Lasioglossum (Dialictus) luciae sp. nov., paratype female **A** lateral habitus **B** dorsal habitus **C** head, frontal view **D** head, lateral view **E** tergum 1, dorsal view.

Females of *L.luciae* and *L.kilpatrickae* are very similar and definitive characters for distinguishing them are not currently known. The gena of *L.luciae* may be more distinctly lineolate (Fig. 5D) and T1 more distinctly coriarious (Fig. 5E), but too few specimens are available of each species to be sure these characters are consistent. Both *L.luciae* and *L.kilpatrickae* are easily distinguished from *L.plumbeum* and *L.sanctivincenti* by absence of punctation on the apical impressed areas of T2, occurring only obscurely on the lateral portions. In contrast, both *L.plumbeum* and *L.sanctivincenti* have distinct, albeit fine punctures across the apical impressed areas of T2.

**Figure 6.**
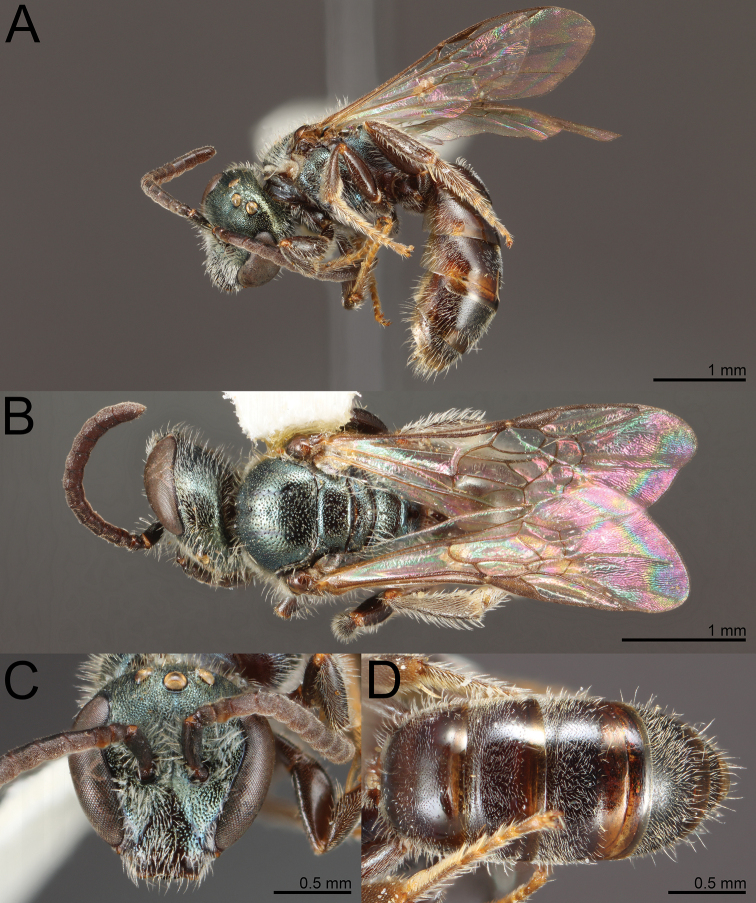
Lasioglossum (Dialictus) luciae sp. nov., holotype male **A** lateral habitus **B** dorsal habitus **C** head, frontal view **D** metasoma, dorsal view.

**Figure 7.**
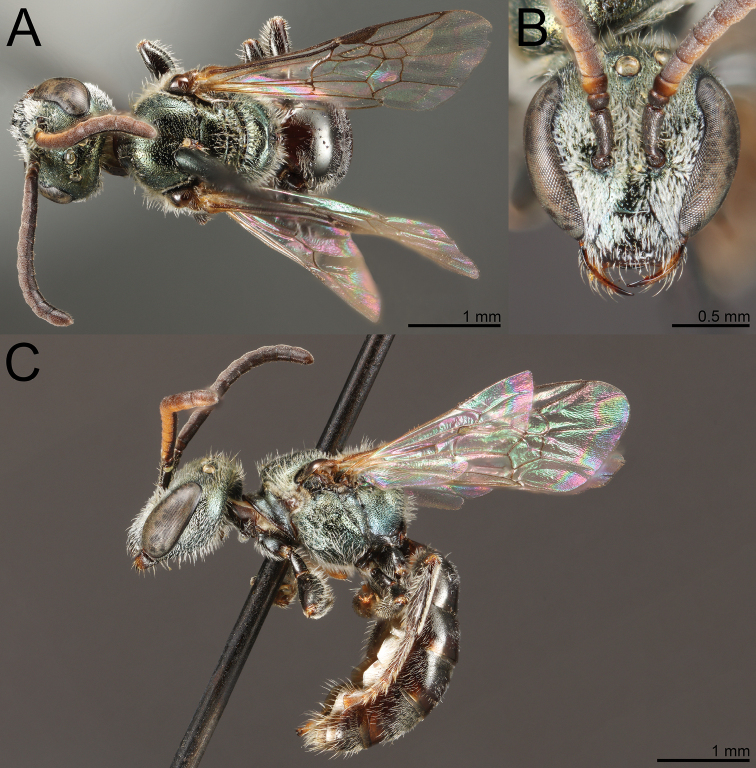
Lasioglossum (Dialictus) kilpatrickae Gibbs, 2016, male **A** dorsal habitus **B** head, frontal view **C** lateral habitus.

The male of *L.luciae* differs from *L.kilpatrickae* by the less abundant tomentum of the face (Fig. 6C), which only weakly obscures the lower paraocular area, more evident microsculpture on the medial portion of the mesoscutum and anterior face of T1, and the relatively dense punctures on T1-T3, which end near the border of the apical impressed area, such that at least two thirds of the segments are densely punctate. *Lasioglossumkilpatrickae* has tomentum obscuring the lower paraocular area and proximal portion of the clypeus (Fig. 7B). The mesoscutum has microsculpture between punctures limited to the anterior portion and is largely polished on the anterior face of T1. Furthermore, the punctation of T1-T3 is weak distally such that nearly half the longitudinal length of the segment is sparsely punctate to impunctate. T1 has a nearly impunctate medial line.

#### Description.

**Female** (*n* = 2). Length 5 mm; head length 1.4 mm; head width 1.4 mm; intertegular distance 1.0 mm; wing length 1.7 mm.

***Colouration*.** Head and mesosoma dull metallic blue-green. Clypeal apex dark brown, base yellow. Labrum reddish brown to orange. Mandible orange with black base and red apex. Flagellum dark brown, F2-F11 orange-brown ventrally. Pronotal lobe reddish brown. Tegula reddish brown. Wing membrane hyaline, veins with subcosta brown to dark brown, otherwise amber. Legs brown, except medio- and distitarsi and portions of metabasitarsus reddish brown. Metasoma blackish brown, apical impressed area reddish brown.

***Pubescence*.** Body with sparse pilosity, dull white to faintly yellowish. Tomentum on gena near eye, pronotum dorsolateral angles and lobe, narrow basolateral patches of T2–T3 and sparsely on T4. Mesoscutal pilosity sparse, erect. Wing setae dark. Acarinarial fan complete, dense. T2 fringes absent, sparse laterally, T3 fringes absent, sparse laterally.

***Surface sculpture*.** Clypeal punctures sparse (IS = 1–4 PD), becoming moderately dense in basal third (IS = 1–2 PD), interspaces polished. Supraclypeal area punctures sparse (IS = 1–3 PD), interspaces weakly imbricate. Paraocular area punctures dense (IS < 1 PD), except near antenna, interspaces imbricate. Frons punctures contiguous. Vertex punctures sparse, interspaces polished. Gena lineolate, postgena lineolate. Tegula punctures obscure. Mesoscutal punctures moderately dense (IS = 1 PD), becoming sparser submedially (IS = 1–1.5 PD) and denser laterad of parapsidal lines (IS ≤ 1 PD), interspaces imbricate, polished laterally; mesoscutellar punctures as in mesoscutum with submedial impunctate area, interspaces imbricate. Metapostnotal rugae strong, anastomosing or subparallel, reaching margin, sculpture imbricate. Pre-episternum rugulose-punctate. Hypoepimeral area densely punctate, interspaces polished. Mesepisternum distinctly punctate. Metepisternum lineolate dorsally, reticulate ventrally. Propodeal lateral face imbricate, sparsely punctate; posterior face imbricate, sparsely punctate. T1 anterior face coriarious; T1 dorsal surface punctures moderately dense (IS = 1–3 PD), absent or very sparse in large apicolateral oval patches, interspaces polished. T2 disc punctures moderately dense (IS = 1–3 PD), interspaces polished, rim impunctate, surface weakly coriarious.

***Structure*.** Face length/width ratio 0.86 (± 0.01 SD). UOD/LOD ratio 1.21 (± 0 SD). Clypeus projecting ~75% below suborbital tangent; apicolateral denticles rounded knobs. Supraclypeal area length/width ratio 2.06 (± 0 SD). Hypostomal carinae parallel. Pronotal angle obtuse. Mesoscutum length/width ratio 0.83 (± 0.01 SD); mesoscutum/mesoscutellum length ratio 2.63 (± 0.1 SD); mesoscutellum/metanotum length ratio 1.66 (± 0.01 SD); metanotum/metapostnotum length ratio 0.75 (± 0.04 SD). Lateral propodeal carinae nearly reaching dorsal margin; oblique carina distinct. Tegula shape ovoid. Forewing with three submarginal cells. Distal hamuli arranged 2-1-2. Inner metatibial spur pectinate, with four branches not including apex of rachis, proximal branch much longer than width of rachis. Metasoma ovoid, apical impressed area medially ~ 1/2 longitudinal length of basal area.

**Male** (*n* = 3). Length 4.4–4.5 mm; head length 1.30–1.35 mm; head width 1.29–1.30 mm; intertegular distance 0.87–0.94 mm. Similar to female with usual sex-associated modifications.

***Colouration*.** Head and mesosoma blue-green. Clypeal apex reddish brown. Labrum reddish brown. Mandible brown, orange apically. Flagellum brown, light brown ventrally. Pronotal lobe reddish brown. Tegula orange. Wing membrane hyaline, veins dark brown. Legs brown with reddish brown tarsi. Metasoma blackish brown, apical impressed areas reddish brown.

***Pubescence*.** Body sparse pilosity, dull white to faintly yellowish. Tomentum moderately dense on lower paraocular area, sparse on clypeus, dense on pronotal lobe. Mesoscutal pilosity thin. Sternal pilosity short (1 OD), moderately plumose, sparse, erect. Wing setae dark, short, sparse.

***Surface sculpture*.** Clypeal punctures dense (IS ≤ 1 PD), interspaces polished. Supraclypeal area punctures sparse (IS = 1–2 PD), interspaces polished. Paraocular area punctures dense (IS ≤ 1 PD), interspaces weakly imbricate around antenna socket, otherwise shiny. Frons punctate-reticulate. Gena punctulate-lineolate, postgena sculpture lineolate. Tegula mostly impunctate. Mesoscutal punctation moderately sparse medially (IS = 1–2 PD), denser laterad of parapsidal lines, interspaces weakly imbricate, polished laterally. Mesoscutellar punctation moderately sparse (IS = 1–2 PD), becoming denser on margins. Metanotum punctate. Metapostnotum incompletely rugulose, margin weakly tessellate. Pre-episternum sculpture punctate. Hypoepimeral area closely punctate (IS ≤ 1 PD), interspaces polished. Mesepisternum distinctly punctate (IS ≤ 1 PD), interspaces shiny. Metepisternum lineolate dorsally, punctate-reticulate ventrally. Propodeal lateral face tessellate-punctate, dorsolateral slope punctate. Propodeal posterior face sculpture tessellate-punctate. T1 anterior face weakly coriarious. T1 dorsal surface evenly punctate (IS = 1–2 PD), interspaces shiny. T2 disc punctures sparse (IS = 1–2.5 PD), interspaces shiny, apical impressed area impunctate, interspaces coriarious.

***Structure*.** Face length/width ratio 0.87–0.88. F1: pedicel length ratio 1.27. F2:F1 length ratio 1.5. Gena narrower than eye. Hypostomal carinae parallel. Pronotal angle obtuse. Mesoscutum length/width ratio 0.82–0.85; mesoscutum/mesoscutellum length ratio 2.44; mesoscutellum/metanotum length ratio 1.78; metanotum/metapostnotum length ratio 0.77. Propodeum lateral carina nearly reaching dorsal margin; oblique carina absent. Tegula ovoid. Forewing with 3 submarginal cells. Metatibial spurs ciliate. Metasoma slender, parallel sided.

#### Etymology.

The specific epithet is derived from the name of the island. Saint Lucia is the only sovereign nation named after a historical woman.

#### Notes.

Males are associated with females in part by the shared head length consistent with patterns seen between *L.dominicense* and *L.kilpatrickae* in Dominica.

### ﻿ Lasioglossum (Dialictus) cf.dominicense

Taxon classificationAnimaliaHymenopteraHalictidae

Gibbs 2016

Fig. 8

Lasioglossum (Dialictus) dominicense Gibbs 2016: 6–11, 42–43.

#### Material examined.

**Saint Lucia** • **Dauphin District** • Louvette trap site, 13.9689, -60.8859, 25–29.VI.2009, leg. M.L. Gimmel and C.A. Maier, UV light trap (1 ♀ MTEC) • Grand Anse trap site, 14.0052, -60.8973, 38 m. 8–17.V.2009, leg. R.C. Winton and E.A. Ivie (1 ♀ MTEC) • **Micoud District** • Escap Community Trail to Fond Bay beach, 13.8324, -60.8986 to 13.8316, -60.893, 46 m to 1 m, 8.V.2009, leg. C.M. Delphia and J.B. Runyon, pan traps (1 ♀ MTEC) • Escap Community, 13.83242, -60.8859, 46 m, 22.V-6.VI.2009, leg. R.C. Winton, Malaise trap (1 ♀ WRME).

**Figure 8.**
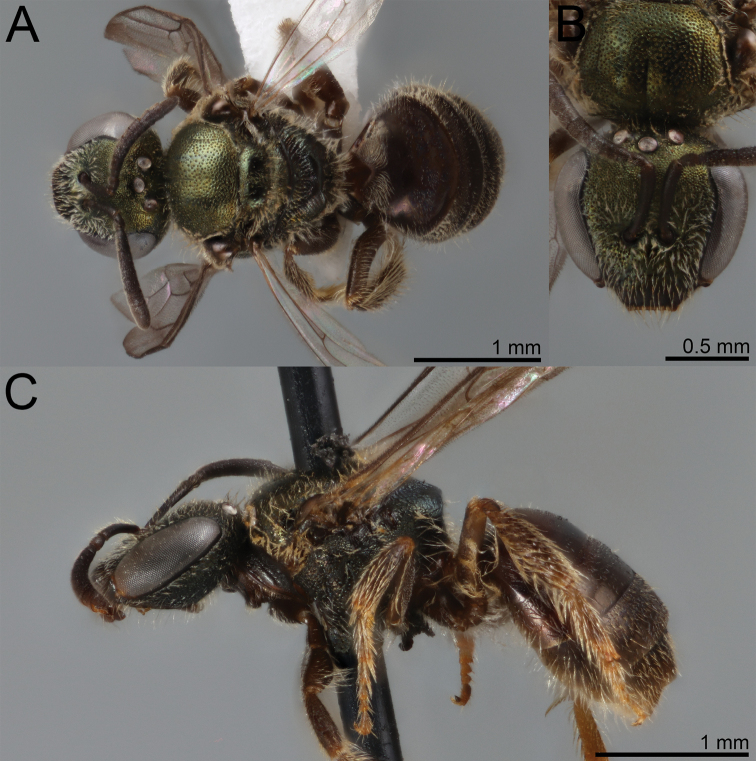
Lasioglossum (Dialictus) cf. dominicense Gibbs, 2016, female **A** dorsal habitus **B** head, frontal view **C** lateral habitus.

#### Notes.

We ascribe the Saint Lucia material to *L.dominicense* without supporting evidence to the contrary. Although there seems to be some pattern of distinct species across islands in the Lesser Antilles, we are unable to confidently differentiate females of *L.dominicense* from Saint Lucia and Dominica at this time. As a lowland species occurring near the beach, it is most consistent with a multi-island distribution. Additional comparative study including males, specimens from Martinique, and molecular data would be useful.

### ﻿Subgenus Habralictellus

Taxon classificationAnimaliaHymenopteraHalictidae

Moure & Hurd, 1982

Habralictellus Moure & Hurd, 1982. Type species: HalictusauratusAshmead 1900, by original designation

### ﻿ Lasioglossum (Habralictellus) delphiae sp. nov.

Taxon classificationAnimaliaHymenopteraHalictidae

http://zoobank.org/D804A754-5BD9-4860-B4C5-793174EFE490

Fig. 9

#### Holotype.

**Saint Lucia**. • Savannes [Bay] Mangrove Res., 13.766, -60.915 [13 45.97 60 54.88], 0–5 m, 3.V.2009, leg. C.M. Delphia (♀ MTEC, to be deposited in the USNM).

**Figure 9.**
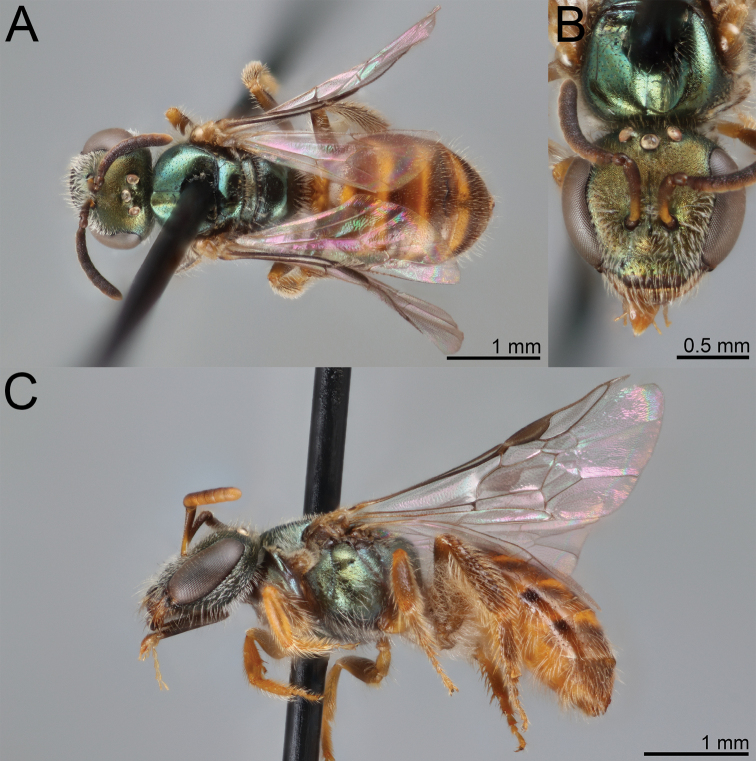
Lasioglossum (Habralictellus) delphiae sp. nov., paratype female **A** dorsal habitus **B** head, frontal view **C** lateral habitus.

#### Paratypes.

**Saint Lucia** • **Micoud District** • Escap Community Fond Bay at beach, [13 83.16 60 89.30], 1 m, 8.V.2009, leg. C.M. Delphia, J.B. Runyon (♀ MTEC).

#### Diagnosis.

*Lasioglossumdelphiae* is easily distinguishable as a member of the subgenus Habralictellus. It has two submarginal cells (1rs-m absent). It closely resembles L. (H.) roseauense from Dominica. *Lasioglossumdelphiae* has the mesoscutellum very weakly sculptured, almost polished with distinct, sparse punctures (mesoscutellum dull, sculpturing stronger, similar to that of mesoscutum in *L.roseauense*) and the metasomal terga have orange bands basally (all dark in *L.roseauense*). There is more yellow on the foreleg of *L.delphiae* than *L.roseauense*, although such colour characters may not be reliable given the limited material available.

#### Description.

**Female** (*n* = 2). Length 4.5 mm; head length 1.1–1.2 mm; head width 1.2–1.3 mm; intertegular distance 0.9–1.04 mm; wing length 1.7–1.8 mm.

***Colouration*.** Head and mesosoma dull metallic golden-green, metapostnotum blue-green. Clypeal apex reddish brown. Labrum reddish brown to orange. Mandible orange with black base and red apex. Scape brown apically, orange basally. Flagellum brown, F3-F11 orange-brown ventrally. Pronotal lobe reddish brown. Tegula amber. Wing membrane hyaline, veins brown. Legs brown, except orange on pro- and mesotrochanters, protibia, protarsi, ventral surface of mesotibia, mesotarsi 2–5, and apices of metafemur and metatibia. Metasomal terga reddish brown with orange patches basally on terga.

***Pubescence*.** Body with sparse pilosity, dull white to faintly yellowish. Tomentum on pronotal dorsolateral angles and lobe. Mesoscutal pilosity sparse erect. Wing setae dark. Acarinarial fan absent, only sparse erect setae on anterior face of T1. Terga with only sparse setae, without apical fringes or basal tomentum.

***Surface sculpture*.** Clypeal punctures sparse (IS = 1–2.5 PD), interspaces weakly imbricate almost polished on apical half, basally tessellate-granular. Supraclypeal punctures sparse (IS = 1–3 PD), interspaces finely reticulate-granular. Paraocular area punctures sparse (IS = 1–2.5 PD), interspaces granular. Frons punctures indistinct, sparse (IS = 1–3 PD). Vertex granular. Gena lineolate, postgena lineolate. Tegula finely punctate on anterior half (IS = 1–2.5 PD), interspaces imbricate, posterior half glabrous. Mesoscutal punctures sparse (IS = 2–3.5 PD), interspaces tessellate; mesoscutellar punctures coarser, sparse (IS = 2–4 PD), interspaces shiny imbricate. Metanotum granular. Metapostnotum transversely lineolate at base, imbricate along apical margins. Preëpisternum tessellate-granular. Hypoepimeral area indistinctly punctate, interspaces tessellate-granular. Mesepisternum indistinct, sparsely punctate (IS = 1–3 PD), interspaces tessellate-granular. Metepisternum lineolate dorsally, imbricate ventrally. Propodeal lateral face tessellate-imbricate, sparsely punctate; posterior face imbricate, sparsely punctate. T1 anterior face polished, dorsally coriarious. T2-T5 sparsely punctate, interspaces coriarious.

***Structure*.** Face length/width ratio 0.77 (0.01 SD). UOD/LOD ratio 1.18 (± 0 SD). Clypeus projecting ~70% below suborbital tangent; apicolateral denticles low rounded knobs. Supraclypeal area length/width ratio 0.7 (± 0.01 SD). Hypostomal carinae parallel. Pronotal angle obtuse. Mesoscutum length/width ratio 0.83 (± 0.04 SD); mesoscutum/mesoscutellum length ratio 2.7 (± 0.09 SD); mesoscutellum/metanotum length ratio 1.98 (± 0.1 SD); metanotum/metapostnotum length ratio 0.57 (± 0.06 SD). Propodeum lateral carinae reaching halfway to dorsal margin; oblique carina absent. Tegula shape ovoid. Forewing with two submarginal cells. Distal hamuli arranged 2-1-2. Inner metatibial spur pectinate, with four branches not including apex of rachis, proximal branch much longer than width of rachis. Metasoma ovoid, apical impressed area medially ~ 1/2 longitudinal length of basal area.

#### Etymology.

The species is named for Casey Delphia for her kind support of JG’s studies of Caribbean bees generally and in appreciation for collecting the specimens above and bringing them to his attention.

#### Notes.

*Lasioglossumdelphiae* was collected from dry forest/beach habitats near the coast (C. Delphia, in litt.).

### ﻿ Lasioglossum (Dialictus) cyaneum

Taxon classificationAnimaliaHymenopteraHalictidae

(Ashmead 1900)

**Figure 11.**
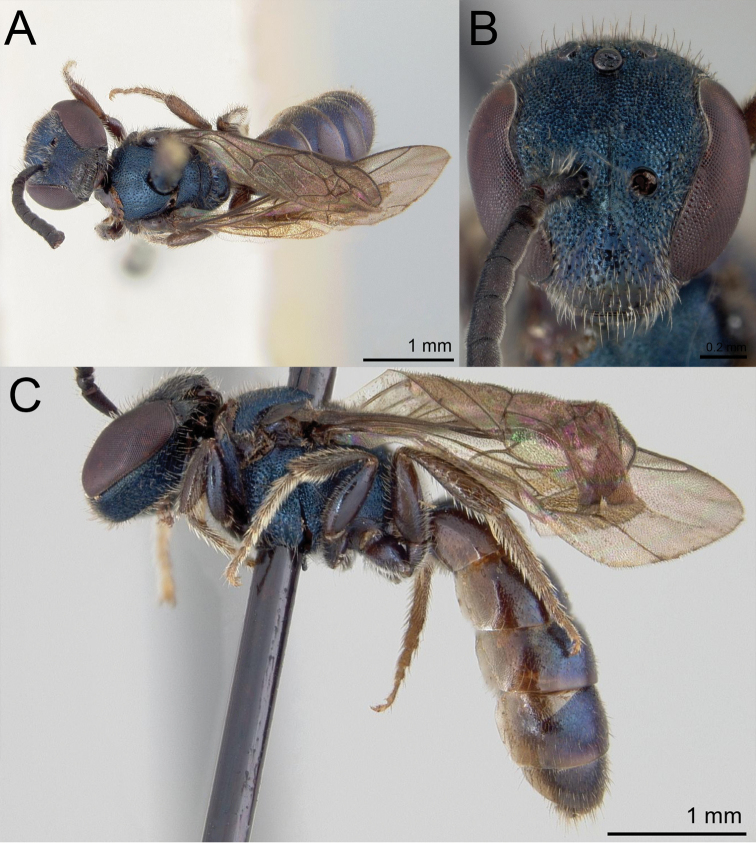
Lasioglossum (Dialictus) cyaneum (Ashmead), syntype male of *Halictuscyaneus* Ashmead **A** dorsal habitus **B** head, frontal view **C** lateral habitus. Images courtesy of the National Museum of Natural History, Smithsonian Institution. https://collections.nmnh.si.edu/search/ento/

**Figure 12.**
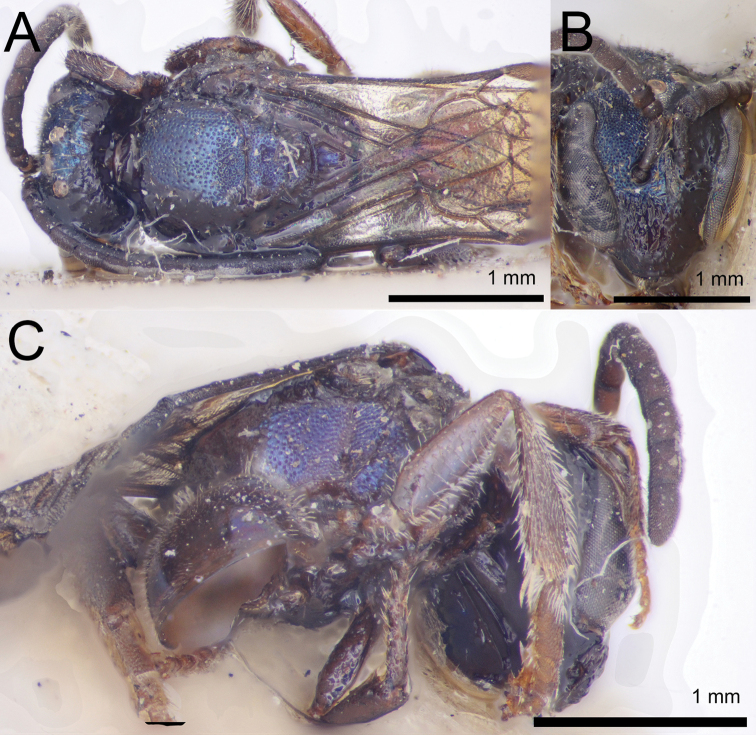
Lasioglossum (Dialictus) cyaneum (Ashmead), syntype male of *Trigonanigrocyanea* Ashmead **A** dorsal habitus **B** head, frontal view **C** lateral habitus. Images courtesy of the Trustees of the Natural History Museum, London (https://creativecommons.org/licenses/by/4.0/). Photographs by David Notton.

**Figure 13.**
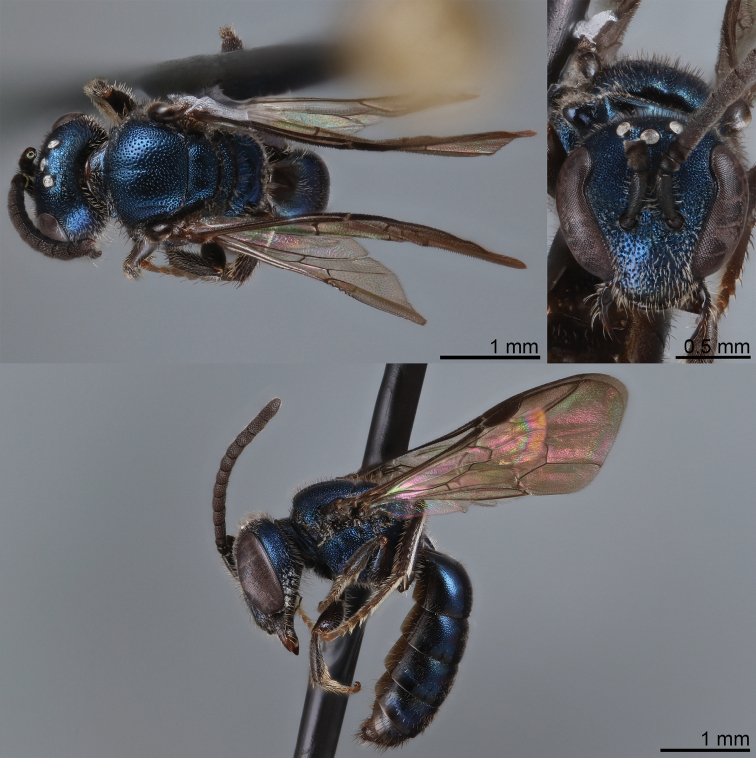
Lasioglossum (Dialictus) cyaneum (Ashmead), male **A** dorsal habitus **B** head, frontal view **C** lateral habitus.

Figs 10 , 11 , 12 , 13

Halictus cyaneus Ashmead (1900: 218–220). Saint Vincent. Syntype males (2) and females (3) (NHMUK, USNM; Figs 10, 11).
Dufourea subcyanea Ashmead (1900: 215). Saint Vincent. Holotype male (NHMUK). Syn. nov.
Trigona nigrocyanea Ashmead (1900: 208). Saint Vincent – Leeward side. Holotype male (NHMUK; Fig. 12). Syn. nov.
Dufourea subcyanea : Ashmead (1900: 303) checklist; Friese (1909: 38) catalogue.
Halictus cyaneus : Ashmead (1900: 304) checklist; Friese (1909: 37) catalogue.
Dialictus cyaneus : Cockerell (1904: 235) taxonomic placement; Moure and Hurd (1987: 98) catalogue; Moure (2007: 848, 849) catalogue.
Dialictus nigrocyaneus : Moure (2007: 852) catalogue.
Dialictus subcyaneus ; Cockerell (1922: 268) taxonomic notes; Sandhouse (1923: 194) checklist; Moure (2007: 855) catalogue; Moure and Hurd (1987: 132) catalogue.
Lasioglossum cyaneum : Gibbs (2016: 6) taxonomic characters.
Trigona nigrocyanea : Ashmead (1900: 299) checklist; Friese (1909: 39) catalogue; Lutz and Cockerell (1920: 499) checklist, type locality.

#### Material examined.

**SVG • Saint Vincent** • Saint Vincent (*Halictuscyaneus* syntypes 1 ♀ 1 ♂ USNM); Saint Vincent (*Dufoureasubcyanea* holotype ♂ NHMUK); Saint Vincent, leeward side (*Trigonanigrocyanea* holotype ♂ NHMUK; from photos) • **St. George Parish** • Majorca Mts., Riley Rd., 13.180694 -61.193556, 366 m, 13.V.2016, leg. Miklasevskaja and Ferrari (1 ♂ WRME) • **St. Patrick Parish** • Cumberland Valley, 17.VI.1977, leg., E.E. Grissell (6 ♂ FSCA).

**Figure 10.** Lasioglossum (Dialictus) cyaneum (Ashmead), syntype female of *Halictuscyaneus* Ashmead **A** dorsal habitus **B** head, frontal view **C** lateral habitus. Images courtesy of the National Museum of Natural History, Smithsonian Institution. https://collections.nmnh.si.edu/search/ento/

#### Taxonomic notes.

*Lasioglossumcyaneum* is structurally similar to *L.plumbeum* and *L.sanctivincenti* but is easily recognisable by the entirely blue body and dark wing venation. The male T1-T6 are blue on the disc and dark reddish brown on the lateral and apical margins. The head is distinctly shorter (female and male face length/head width = 0.82–0.85) than *L.plumbeum* (male face length/head width = 0.87–0.90). Both *Dufoureasubcyanea* and *Trigonanigrocyanea* were described from single males in the same publication with *Halictuscyaneus*. The former differs from *L.cyaneum* only in the absence of vein 1rs-m, leading to two submarginal cells rather than three. Loss of this vein is relatively common in L. (Dialictus) (Gibbs 2010b; Scarpulla 2018; see also *L.gemmeum* below), which led to the synonymy of the genus-group names *Dialictus* and *Chloralictus* (Mitchell 1960). The holotype of *Trigonanigrocyanea* is glued to the side of a card and has most of the metasoma missing. It is very evidently a Lasioglossum (Dialictus). The first tergum is intact and shows distinct metallic reflections consistent with *L.cyaneum*. Ashmead (1900) describes the abdomen as ‘rufous, black at base only’, but cannot be verified with most of the metasoma missing. In other respects, the holotype matches well with *L.cyaneum*, including the relatively smooth metapostnotum between carinulae.

### ﻿ Lasioglossum (Dialictus) plumbeum

Taxon classificationAnimaliaHymenopteraHalictidae

(Ashmead 1900)

Figs 14 , 15 , 16

**Figure 14.**
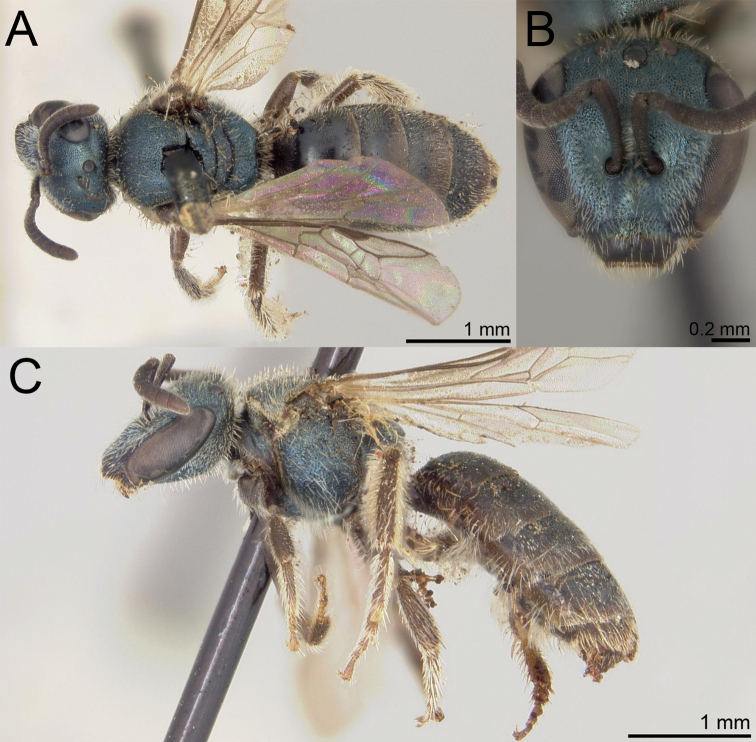
Lasioglossum (Dialictus) plumbeum (Ashmead), syntype female of *Halictusplumbeus* Ashmead **A** dorsal habitus **B** head, frontal view **C** lateral habitus. Images courtesy of the National Museum of Natural History, Smithsonian Institution. https://collections.nmnh.si.edu/search/ento/

Halictus plumbeus Ashmead (1900: 218, 220). Saint Vincent. Syntype males and females (NHMUK, USNM; Fig. 14). Examined.
Halictus plumbeus : Ashmead (1900: 304) checklist; Friese (1909: 37) catalogue; Cockerell (1915: 9) taxonomic note; Cockerell (1938: 280, 281) taxonomic notes.
Halictus (Chloralictus) plumbeus : Sandhouse (1924: 4) identification key; Cockerell (1937: 113) taxonomic notes.
Dialictus plumbeus : Moure and Hurd (1987: 124) catalogue; Moure (2007: 853) catalogue.
Lasioglossum plumbeum : Gibbs (2016: 6, 15) taxonomic notes.

#### Material examined.

**SVG • Saint Vincent** • St. Vincent, leg. H.H. Smith (*Halictusplumbeus* syntypes 1 ♀ NHMUK, 1 ♀ USNM) • St. Vincent (Windward side), leg. H.H. Smith (2 ♀ USNM) • **Charlotte Parish** • Belair Mespo Peruvian Vale Rd., 13.173417 -61.151111, 71 m, 13.V.2016, leg. Miklasevskaja and Ferrari (1 ♂ WRME) • Fancy, 1 km S of Windward hwy. 13.380122 -61.170588, 55 m, 18.V.2016, leg. Miklasevskaja and Ferrari (1 ♂ WRME) • Greiggs, Charlotte Mtn.,13.222417 -61.173361, 478 m, 14.V.2016, leg. Miklasevskaja and Ferrari (1 ♂ WRME) • **St. Andrew Parish** • Vermont Trail Rd., 13.201639 -61.241333, 114 m, 15.V.2016, leg. Miklasevskaja and Ferrari (1 ♀ WRME) • **St. David Parish** • Cumberland Way, 19.IX.1991, leg. R.E. Woodruff, near beach (1 ♀ 1 ♂ FSCA) • Wallilabou, 14.X.1991, leg. R.E. Woodruff, day catch (10 ♀ 2 ♂) • **St. George Parish** • Cane Hall, 22.IX.1991, leg. R.E. Woodruff, sweeping (4 ♀ 9 ♂ FSCA) • Cane Hall, Rick’s Apts., 17.IX.1991, leg. R.E. Woodruff, vacant lot (1 ♀ FSCA) • Rivulet Agr. Sta. 10–15.X.1991, leg. R.E. Woodruff, Malaise trap (3 ♀ FSCA); 27–30.IX.1991, leg. R.E. Woodruff, Malaise trap (1 ♀ 1 ♂ FSCA) • Majorca Mts., Riley Rd., 13.180694 -61.193556, 366 m, 13.V.2016, leg. Miklasevskaja and Ferrari (1 ♀ WRME) • **St. Patrick Parish** • Cumberland Valley, 17,VI.1977, leg. E.E. Grissell (11 ♀ 21 ♂ FSCA) • Hermitage Forestry Cottage, 11–13.X.1991, leg. R.E. Woodruff, day catch (2 ♀ 1 ♂ FSCA) • Rutland Vale, 1 km N on Leeward Hwy., 13.218727 -61.270954, 60 m, 19.V.2016, leg. Miklasevskaja and Ferrari (1 ♀ WRME) • **Grenadines** • Bequia, Industry, 24.IX.1991, leg. R.E. Woodruff (1 ♂ FSCA).

#### Notes.

*Lasioglossumsanctivincenti* is quite similar to *L.plumbeum*. The most striking difference is the darker blue colour of the head and mesosoma of *L.plumbeum*. *Lasioglossumsanctivincenti* has a shorter head (face length/head width ratio = 0.82 SD 0.02) than *L.plumbeum* (0.86 SD 0.01). Mesoscutal puncture density is subtly different between the two species. In *L.sanctivincenti* punctures laterad of the parapsidal line are dense, but distinctly separated. These are nearly reticulate in *L.plumbeum*, without clear interspaces. Immediately mesad of the parapsidal line, *L.sanctivincenti* has distinctly separated punctures (IS ≤ 1 PD), but these are denser in *L.plumbeum* (IS ≤ 0.5 PD). Ashmead’s (1900) original measurements suggest that *L.santivincenti* is larger (4–5.5 mm) than *L.plumbeum* (3.5–4.5 mm). In Sandhouse’s (1924) key, they separate at couplet 43 based on size and Cockerell (1938) also refers to the smaller size of *L.plumbeum*. However, this may be an artefact of H.H. Smith’s original sample as more recently collected specimens of *L.plumbeum* include a large size range (> 5 mm) overlapping with that of *L.sanctivincenti*. The size variation in *L.plumbeum* may be an indication of weakly defined social castes in *L.plumbeum*, which is a common feature of eusocial halictines (Michener 1990).

**Figure 15.**
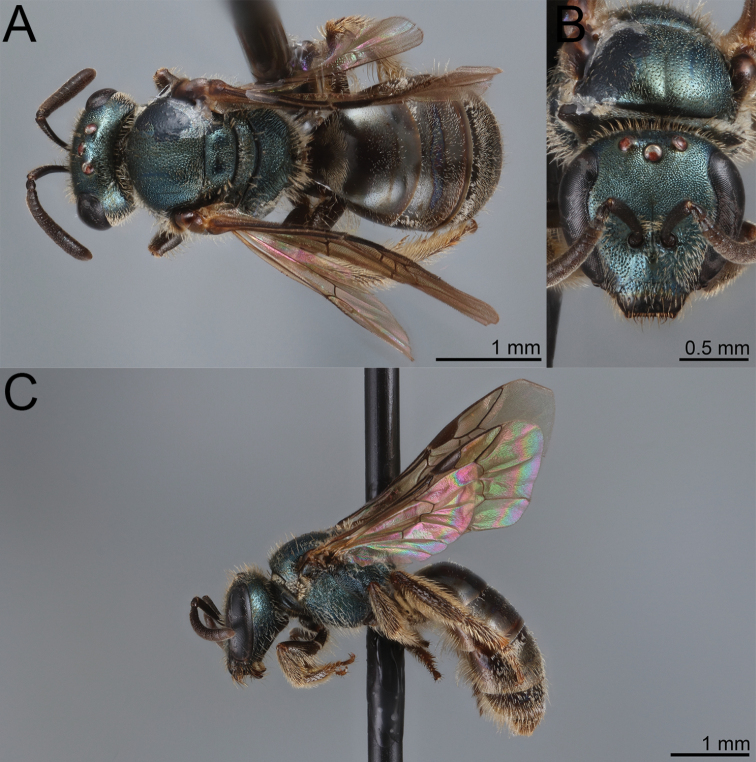
Lasioglossum (Dialictus) plumbeum (Ashmead), female **A** dorsal habitus **B** head, frontal view **C** lateral habitus.

**Figure 16.**
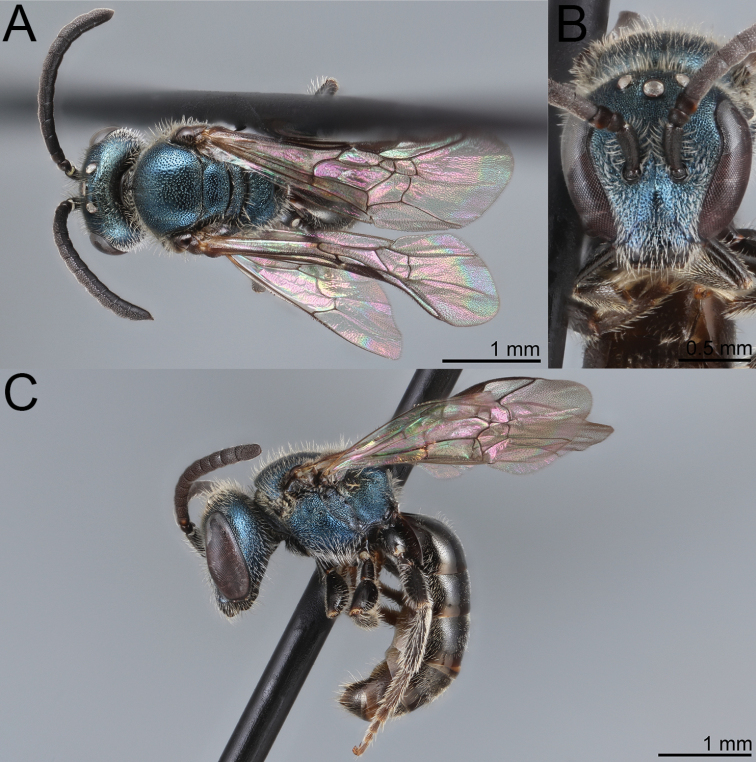
Lasioglossum (Dialictus) plumbeum (Ashmead), male **A** dorsal habitus **B** head, frontal view **C** lateral habitus.

### ﻿ Lasioglossum (Dialictus) sanctivincenti

Taxon classificationAnimaliaHymenopteraHalictidae

(Ashmead 1900)

Figs 17 , 18 , 19

**Figure 17.**
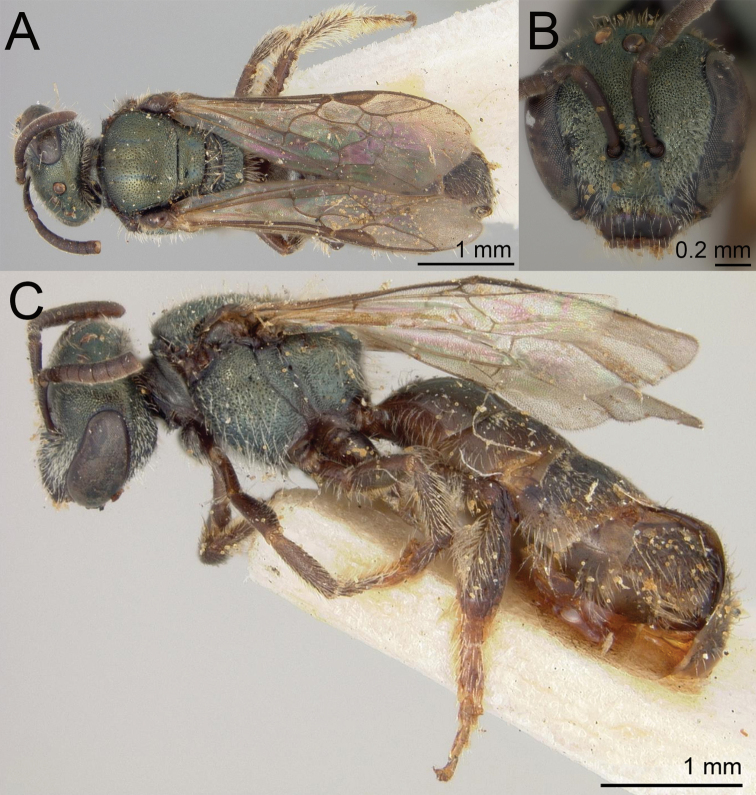
Lasioglossum (Dialictus) sanctivincenti (Ashmead), syntype female of *Halictussanctivincenti* Ashmead **A** dorsal habitus **B** head, frontal view **C** lateral habitus. Images courtesy of the National Museum of Natural History, Smithsonian Institution. https://collections.nmnh.si.edu/search/ento/

Halictus sancti-vincenti Ashmead (1900: 218–220). Grenada – St. George’s; Mount Gay Estate (Leeward side), Saint Vincent. Syntype males and females (NHMUK, USNM; Fig. 17).
Halictus santivincent : Friese (1909: 37) catalogue [sic].
Halictus sancti-vincenti : Ashmead (1900: 304) checklist; Cockerell (1938: 280, 281) taxonomic notes.
Halictus (Chloralictus) sanctivincenti : Sandhouse (1924: 5) emendation, identification key; Cockerell (1937: 113) taxonomic notes.
Dialictus sanctivincenti : Moure and Hurd (1987: 128, 129) catalogue, possible synonymy; Moure (2007: 854) catalogue.
Lasioglossum sanctivincenti : Gibbs (2016: 6, 11) taxonomic notes.

#### Material examined.

**SVG • Grenadines** • Canoun Island, 7.X.1991, leg. R.E. Woodruff (3 ♀ FSCA). Bequia Island, 1966–VI.1967, leg. Badger (1 ♀ UNSM). **Grenada** • Carricou Island, Hillsborough, the Sands Guest House, 1.III.1990, leg. R.E. Woodruff (1 ♀ FSCA) • **St. Andrew Parish** • Grand Etang, XI.1950, leg. N.L.H. Krauss (1 ♀ USNM) • **St. George Parish** • Mount Gay Est., leg. H.H. Smith (*Halictussanctivincenti* syntype 1 ♂ USNM) • St. Georges (Leeward side), leg. H.H. Smith (2 ♀ USNM, *Halictussanctivincenti* syntype 1 ♀ NHMUK) • St. Georges, XI.1950, leg. N.L.H. Krauss (11 ♀ USNM) • **St. John Parish** • Woodford, 5.VIII.1963, leg. O.S. Flint (1 ♀ USNM).

#### Notes.

The syntype series of *L.sanctivincenti* is divided between Grenada and St. Vincent (Ashmead 1900), which are islands separate by approximately 100 km. However, there are 22 intermediary islands in the Grenadine Island chain, so the maximum distance between landmasses is an order of magnitude less. Despite the name, *L.sanctivincenti* does not seem common on St. Vincent. In fact, all the specimens examined above belong are from islands to the south. To date, *L.sanctivincenti* and *Habralictusinsularis*Smith-Pardo 2009 are the only halictid bees known from Grenada. Cockerell (1937) records *L.sanctivincenti* from Barbados, 160 km east of St. Vincent, however, his description of the darker colour and ‘mesothorax highly polished’ do not seem consistent with the syntype series of *L.sanctivincenti*.

**Figure 18.**
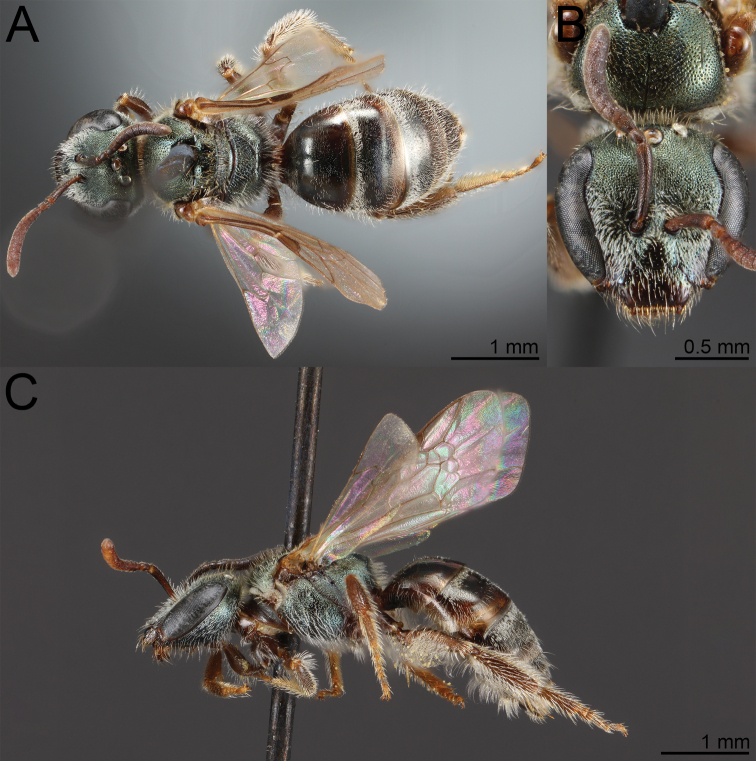
Lasioglossum (Dialictus) sanctivincenti (Ashmead), female **A** dorsal habitus **B** head, frontal view **C** lateral habitus.

**Figure 19.**
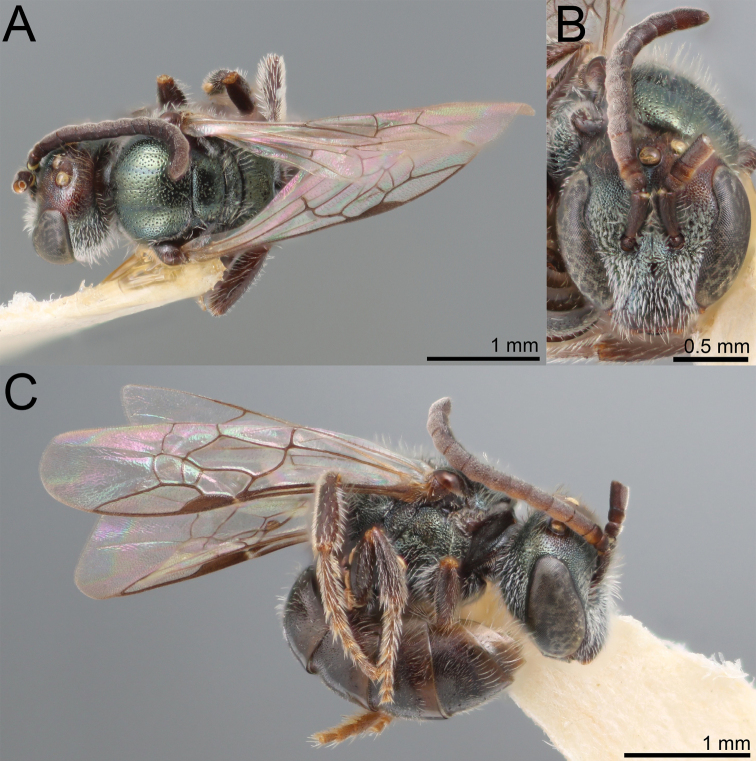
Lasioglossum (Dialictus) sanctivincenti (Ashmead), male **A** dorsal habitus **B** head, frontal view **C** lateral habitus.

### ﻿ Lasioglossum (Dialictus) gemmeum sp. nov.

Taxon classificationAnimaliaHymenopteraHalictidae

http://zoobank.org/61DF8422-3F04-4E4D-A201-4107060FE9B4

Figs 20 , 21

#### Holotype.

♀. Saint Vincent, St. George Parish, 5–10.X.1991, leg. R.E. Woodruff, Malaise trap (FSCA).

#### Paratypes.

**SVG • Saint Vincent• St. George Parish** • Rivulet Agr. Sta., 27–30-IX-1991, leg. R.E. Woodruff, Malaise trap (1 ♂); 5–10-X-1991, leg. R.E. Woodruff, Malaise trap (2 ♀ FSCA) • “24 // W. Indies / 99-331 // Dialictus ­not gemmatus det G.C. Eickwort” (1 ♀ NHMUK). One leg, both forewings and one hind wing missing. “69 // W. Indies 99-331 // Halictusgemmatus Smith Ashm // Dialictus ­not gemmatus det G.C. Eickwort” (NHMUK). In good condition, two submarginal cells in both wings (1 ♀ NHMUK) • St Vincent, Majorea, VIII.1972 (2 ♂ SEMC).

**Figure 20.**
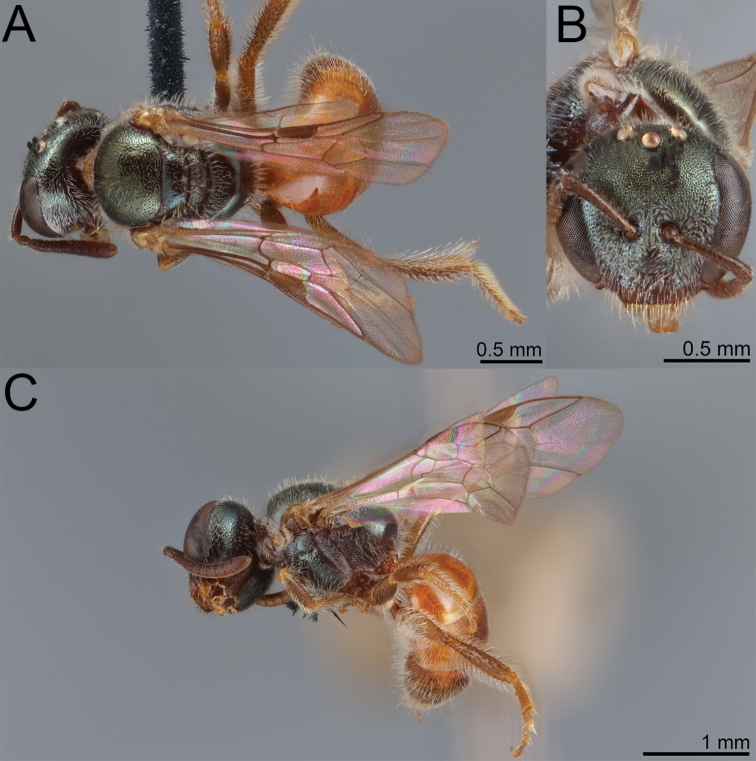
Lasioglossum (Dialictus) gemmeum sp. nov., holotype female **A** dorsal habitus **B** head, frontal view **C** lateral habitus.

*Halictusgemmatus*: Ashmead (1900: 218, 219, 303) key, distribution record (in part); Friese (1909: 37) catalogue. Non *gemmatus* Smith, 1853.

*Dialictusgemmatus*: Moure and Hurd (1987: 101) catalogue (in part); Moure (2007: 849) catalogue (in part). Non *gemmatus* Smith, 1853.

#### Diagnosis.

Females of *L.gemmeum* are easily recognised by their orange-red metasoma and small size (~ 3.5 mm long). No other L. (Dialictus) in the Caribbean is known to have such a brightly coloured metasoma, although some L. (Habralictellus) do. Males can be distinguished from other SVGL. (Dialictus) by the elongate (1.5–2 MOD), pectinate setae on S5-S6. Other SVGL. (Dialictus) have short (1 MOD), simple setae on S5-S6, which contrast with plumose setae on preceding sternites.

**Figure 21.**
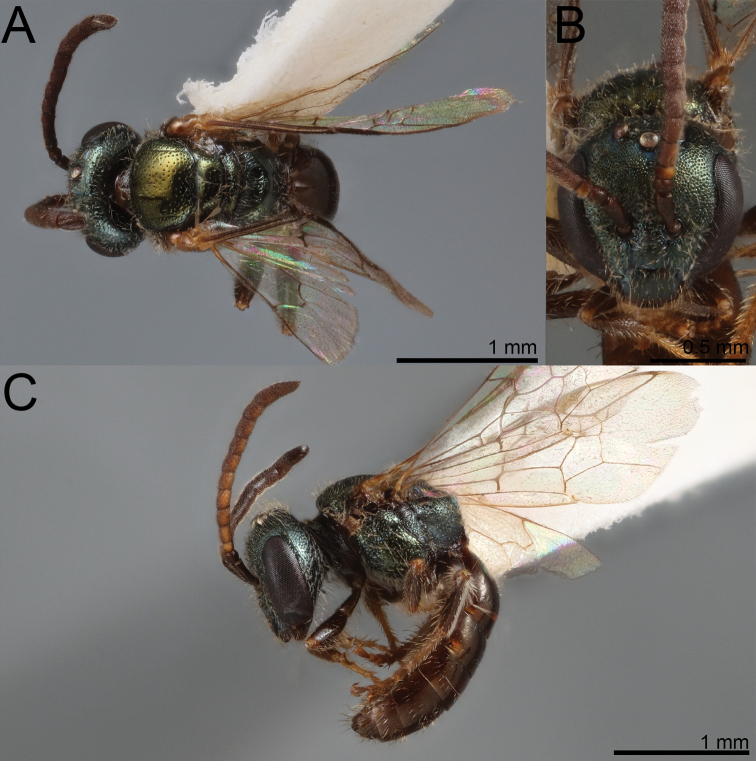
Lasioglossum (Dialictus) gemmeum sp. nov., paratype male **A** dorsal habitus **B** head, frontal view **C** lateral habitus.

#### Description.

**Female** (*n* = 5). Length 3.3–3.6 mm; head length 1.03–1.11 mm; head width 1.08­–1.19 mm; intertegular distance 0.71–0.84 mm; wing length 1.38–1.60 mm.

***Colouration*.** Head and mesosoma dull metallic blue-green to golden-green, except as follows. Labrum reddish brown. Mandible yellow-orange with brown base and red apex. Clypeal apex dark brown. Antenna dark brown, flagellum with ventral surface reddish brown. Pronotal lobe yellow-orange. Tegula amber. Wing membrane hyaline with dark setae, venation pale brown. Legs amber-brown. Metasomal terga orange.

***Pubescence*.** Dull white. Relatively sparse erect setae throughout, without tomentum, except on gena near eye, pronotal dorsolateral angle and lobe. Metasomal T1 with fan virtually absent, no erect setae medially. T2 without apical fimbriae, T3–T4 with only sparse fine setae on apical impressed areas. Scopa well developed on hind leg and metasomal sterna.

***Surface sculpture*.** Face imbricate, punctation moderately fine. Clypeal punctation moderately sparse (IS = 1–s PD), denser proximally (IS = 1 PD), surface smooth distally. Supraclypeal area with punctures moderately sparse (IS = 1–2 PD), weakly imbricate in centre. Lower paraocular area punctation dense (IS ≤ PD). Upper paraocular area and frons reticulate-punctate (IS < PD). Ocellocular area punctate (IS ≤ PD). Gena and postgena punctate-imbricate, sculpturing weak on postgena. Mesoscutum weakly imbricate, polished submedially; punctation moderately coarse, dense laterad of parapsidal lines, posterior portion (IS < PD), sparsest submedially (IS = 1–2 PD), mesoscutellum similar with submedial impunctate area (IS = 1–3 PD). Metanotum finely punctate. Preëpisternum finely reticulate rugulose. Hypoepimeral area finely punctate. Mesepisternum below scrobe punctate (IS ≤ d), polished. Metepisternum dorsal 1/3 lineolate, ventral portion reticulate-imbricate. Metapostnotum medially with irregular carinulae reaching 2/3 distance to imbricate posterior margin, dorsolateral slope imbricate. Propodeum posterior and lateral surfaces weakly imbricate. Metasomal terga polished, finely coriarious basally, weakly coriarious on apical impressed margin of T3; punctation sparse (IS = 2–3 PD) on basal half, indistinct, sparser on apical impressed areas, T1-T2 apical impressed areas nearly impunctate. Metasomal sterna coriarious and finely, sparsely punctate (IS = 2–4 PD).

***Structure*.** Face relatively short (length/width ratio = 0.82 ± 0.01 SD). Eyes weakly convergent below (UOD/LOD ratio = 1.29 ± 0.19 SD). Clypeus 2/3 below suborbital tangent, apicolateral denticles low rounded knobs. Gena narrower than eye. Hypostomal carinae subparallel. Pronotal dorsolateral angle obtuse. Pronotal ridge rounded, interrupted by sulcus. Mesoscutum length/width ratio 0.82 (± 0.02 SD); mesoscutum/mesoscutellum length ratio 2.72 (± 0.2 SD); mesoscutellum/metanotum length ratio 1.75 (± 0.06 SD); metanotum/metapostnotum length ratio 0.64 (± 0.03 SD). Tegula ovoid. Submarginal cells two or three, veins 1r-sm, 2rs-m and 2m-cu distinctly weak. Distal hamuli arranged 2-1-2. Inner metatibial spur pectinate, with two or three branches not including apex of rachis, proximal branch much longer than width of rachis. Metapostnotum narrowly rounded onto posterior propodeal surface. Propodeum with lateral carina reaching 1/2 distance dorsal margin; oblique carina indistinct. Metasoma ovoid, T2–T4 impressed areas medially ~ 1/2 longitudinal length of basal area.

**Male** (*n* = 3). Length 3.3–3.5 mm; head length 1.00–1.08 mm; head width 1.00–1.11 mm; intertegular distance 0.67–0.79 mm. Similar to female with usual sex-associated modifications.

***Colouration*.** Head and mesosoma green to golden green. Clypeal apex reddish brown. Labrum reddish brown. Mandible brown, orange apically. Flagellum reddish brown, sometimes orange ventrally. Pronotal lobe reddish brown to orange. Tegula orange. Wing membrane hyaline, veins brown to dark brown. Legs reddish brown with femur-tibia joints, base and apex of tibiae, and tarsi orange. Metasoma reddish brown.

***Pubescence*.** Body sparse pilosity, dull white to faintly yellowish. Tomentum moderately dense on lower paraocular area, sparse on clypeus, dense on pronotal lobe. Mesoscutal pilosity thin. Sternal pilosity short (1.0–1.5 OD), densely plumose, dense, erect. Wing setae dark, short, sparse.

***Surface sculpture*.** Clypeal punctures dense (IS ≤ 1 PD), interspaces polished. Supraclypeal punctures sparse (I = 1–2 PD), interspaces polished. Paraocular area punctures dense (IS ≤ 1 PD), interspaces shiny. Frons punctate-reticulate. Gena punctate-imbricate, postgena sculpture punctate-imbricate. Tegula mostly impunctate. Mesoscutal punctation sparse (IS = 1–3 PD), becoming dense marginally (IS = 1–1.5 PD), interspaces shiny. Mesoscutellar punctation sparse (IS = 1–2 PD). Metanotum punctate. Metapostnotum with incomplete carinulae, margin shiny to weakly imbricate. Pre-episternum sculpture punctate. Hypoepimeral area distinctly punctate IS ≤ 1 PD), interspaces polished. Mesepisternum distinctly punctate (IS ≤ 1 PD), interspaces shiny. Metepisternum lineate dorsally, weakly rugulose ventrally. Propodeal lateral face weakly imbricate-punctate, dorsolateral slope punctate. Propodeal posterior face sculpture polished-punctate. T1 anterior face polished. T1 dorsal surface sparse (IS = 2–6 PD), interspaces shiny. T2 disc punctures sparse (IS = 1–2.5 PD), failing well before premarginal line, interspaces shiny, apical impressed area impunctate, interspaces shiny.

***Structure*.** Face length/width ratio 0.84 (± 0.03 SD). F1: pedicel length ratio 0.77–1.00. F2:F1 length ratio 1.76–1.89. Gena narrower than eye. Hypostomal carinae parallel. Pronotal angle obtuse. Mesoscutum length/width ratio 0.0.8 (± 0.02 SD); mesoscutum/mesoscutellum length ratio 2.51 (± 0.03 SD); mesoscutellum/metanotum length ratio 2.04 (± 0.25 SD); metanotum/metapostnotum length ratio 0.59 (± 0.07 SD). Propodeum lateral carina nearly halfway to dorsal margin; oblique carina absent. Tegula ovoid. Forewing with two or three submarginal cells. Metatibial spurs ciliate. Metasoma slender, parallel sided.

#### Etymology.

The specific epithet is a Latin adjective in the nominal singular meaning glittering.

#### Taxonomic notes.

Ashmead (1900) recorded three specimens of this species as *Halictusgemmatus* from the Leeward and Windward sides of St. Vincent. Comparison of two of his specimens to the type of *H.gemmatus* from Jamaica, indicated that they were quite distinct. Both specimens have labels attached from George Eickwort indicating it is not *gemmatus*. One of these is missing both forewings and the other has vein 1rs-m missing in both wings. The three other females have 1rs-m present, but the single male paratype has 1rs-m absent in the left wing and present in the right wing. *Lasioglossumgemmatum* is a member of the *gemmatum* species complex (also known as the *parvum* or *tegulare* species complex; Ellis 1914; Gibbs 2009, 2018), but *L.gemmeum* does not appear to be a member of this group.

### ﻿ Lasioglossum (Habralictellus) auratum

Taxon classificationAnimaliaHymenopteraHalictidae

(Ashmead 1900)

Fig. 22

**Figure 22.**
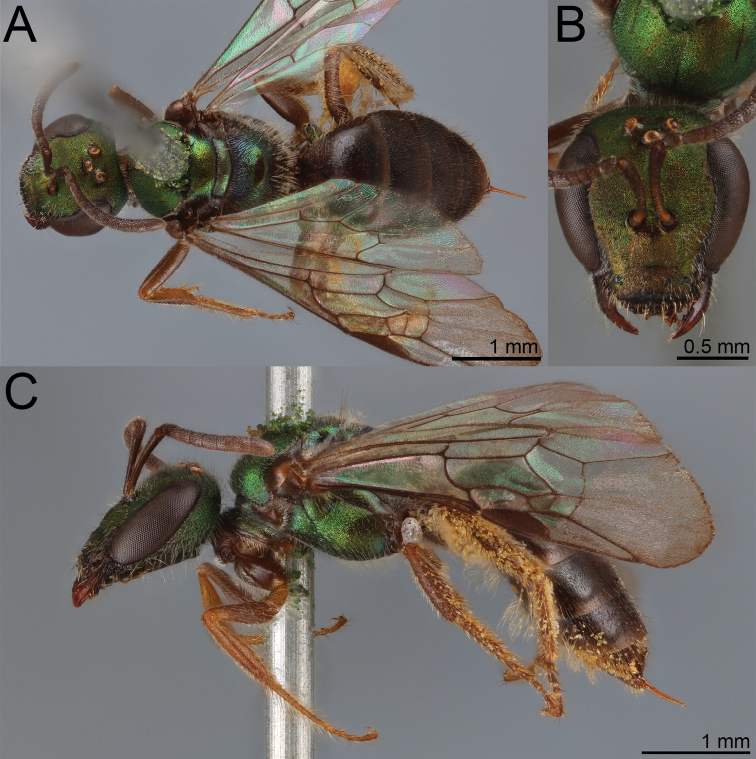
Lasioglossum (Habralictellus) auratum (Ashmead), syntype female of *Halictusauratus* Ashmead **A** dorsal habitus **B** head, frontal view **C** lateral habitus.

Halictus auratus Ashmead 1900: 220. Saint Vincent – windward side (1500 ft.), seven female and one male syntypes (NHMUK, USNM; Fig. 22).
Halictus auratus : Friese (1909: 37) catalogue; Cockerell (1913: 104) taxonomic notes; Crawford (1914: 133) comparative notes; Moure and Hurd (1987: 205) catalogue (unplaced taxon).
Habralictellus auratus : Moure and Hurd (1982: 46) taxonomy, genus description; Moure (2007: 858) catalogue.
Lasioglossum (Dialictus) auratum : Michener (2000: 361) genus-group synonymy.
Lasioglossum (Habralictellus) auratum : Gibbs (2016: 17, 2018: 43) taxonomic notes; Genaro (2021: 14) taxonomic notes, checklist.

#### Material examined.

**SVG • Saint Vincent** • Saint Vincent (windward side), 1500 ft. (*Halictusauratus* syntypes 1 ♀ NHMUK, 3 ♀ USNM).

#### Taxonomic notes.

*Lasioglossumauratum* is the type species of *Habralictellus*, a genus group that has fluctuated between treatments as a genus (Moure and Hurd 1982; Engel 2001b), subgenus of *Lasioglossum* (Gibbs 2016, 2018; Genaro 2021), or a synonym of L. (Dialictus) (Michener 2000; Genaro 2001b, 2016). Preliminary molecular phylogenetic data suggests L. (Habralictellus) is distinct from L. (Dialictus) (Gibbs 2018). The differences in size, sculpturing, and male genitalia evident in described L. (Habralictellus) suggests that it may not be monophyletic (Gibbs 2018).

**Figure 23.**
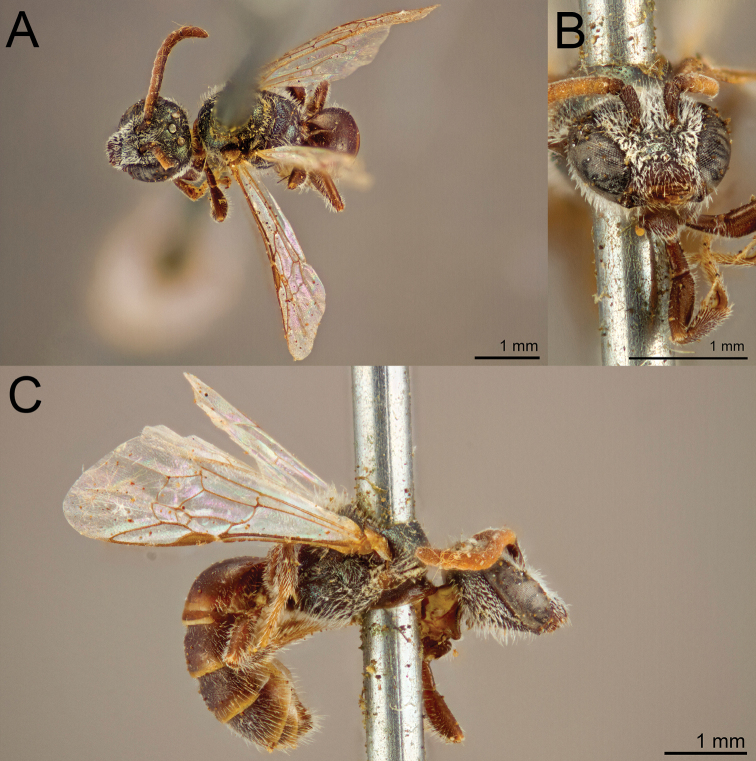
Lasioglossum (Dialictus) minutum (Fabricius), syntype male of *Hylaeusminutus* Fabricius **A** dorsal habitus **B** head, oblique frontal-ventral view **C** lateral habitus. Images courtesy of the Natural History Museum of Denmark. Photographs by Mikkel Høegh Post. http://www.daim.snm.ku.dk/search-in-types

### ﻿Key to *Lasioglossum* of Saint Vincent and the Grenadines

**Table:** 

1	Head and mesosoma brilliant metallic golden-green (Fig. 22); mesoscutum granular with extremely fine and indistinct punctation; subgenus >Habralictellus	***L.auratum***
–	Head and mesosoma dull metallic golden-green to blue; mesoscutum imbricate to weakly polished with relatively coarse and distinct punctation; subgenus Dialictus	**2**
2	Metasoma dark metallic blue (Figs 10–13); wings relatively dark	***L.cyaneum***
–	Metasoma brown to orange; wings relatively pale	**3**
3	Female	**4**
–	Male	**6**
4	Metasoma orange-red (Fig. 20); tegula pale orange	***L.gemmeum***
–	Metasoma brown; head longer; tegula reddish brown to dark brown	**5**
5	Head and mesosoma blue (Figs 14, 15); face relatively long (length/width ratio = 0.86 SD 0.01); punctation near parapsidal line very dense (IS < 0.5 PD); mesepisternum with interspaces shiny due to weak microsculpture	***L.plumbeum***
–	Head and mesosoma golden green (Figs 17, 18); face relatively short (length/width ratio = 0.82 SD 0.02); punctation near parapsidal line sparser (IS ≤ 1 PD); mesepisternum with interspaces dull due to distinct microsculpture	***L.sanctivincenti***
6	Mesoscutum disc shiny, punctation sparse (Fig. 21); tegula pale orange; S5-S6 with long (1.5–2 MOD), pectinate setae	***L.gemmeum***
–	Mesoscutum disc duller, punctation denser (Figs 16, 19); tegula reddish brown to dark brown; S5-S6 with short (1 MOD), simple setae	**7**
7	Head and mesosoma blue; face relatively long (length/width ratio = 0.86)	***L.plumbeum***
–	Head and mesosoma golden green; face relatively short (length/width ratio = 0.82)	***L.sanctivincenti***

### ﻿ Lasioglossum (Dialictus) minutum

Taxon classificationAnimaliaHymenopteraHalictidae

(Fabricius 1798)

Fig. 23

Hylaeus minutus Fabricius 1798: 272. Americaeinsulus. Syntype ♂ (Natural History Museum of Denmark).
Prosopis minuta : Dalla Torre (1896: 27) catalogue; Fabricius (1804: 295) redescription.
Dialictus (Chloralictus) minutus : Moure (1960a: 101) redescription, taxonomic status, distribution; Moure (1960b: 76) redescription, taxonomic status.
Lasioglossum (Evylaeus) minutum : Ebmer (1974: 117, 122) taxonomic status, nomenclature, distribution.
Dialictus minutus : Moure and Hurd (1987: 114, 128, 129) taxonomic status, nomenclature, distribution; Moure (2007: 851) catalogue.

#### Taxonomic notes.

The distribution and identity of *L.minutum* remains in doubt. Fabricius (1798) did not specify the number of specimens examined, but a single male type is known. Moure (1960a) examined this type of *Hylaeusminutus* Fabricius and transferred it to Dialictus (Chloralictus). The type locality is “*Americaeinsulus*”, clarified subsequently to be “*Americaemeridionalisinsulus*” (Fabricius 1804). Moure (1960b) thought it was from St. Vincent. Moure and Hurd (1987) considered it a possible senior synonym of *L.sanctivincenti*. However, Ebmer (1974) suggests that the specimen may be from St. Thomas in the Virgin Islands, as the underside of the label read “S. Thomae”. The latter locality information may not be a reliable indication of the specimen’s original collection, but rather the shipping origin to Denmark (L. Vilhelmsen, *pers. comm.*). Photographs of the specimen were examined (Fig. 23), and it seems consistent with *L.sanctivincenti*. Without certainty of its island of origin or physical examination of the holotype of *L.minutum* a formal synonymy seems premature.

The nomenclature of *L.minutum* is somewhat confusing as discussed by earlier authors (Ebmer 1974). Moure and Hurd (1987) considered it preoccupied by Schrank (i.e., *Apisminuta* Schrank 1781). However, Ebmer (1974) considered Schrank’s bee to be a *Hylaeus*, although Warncke (1975) disagreed. Unless Schrank’s bee can be assigned to *Lasioglossum*, it cannot be considered a senior secondary homonym. Two definite cases of secondary homonymy exist, one of which has not been previously resolved. Kirby (1802: 61–62) described *Melittaminuta*, which he attributes to Schrank. However, Kirby is typically credited with authorship (Blüthgen 1921; Ebmer 1974) since he acknowledged key differences between his bee and Schrank’s and doubted that they were the same. Kirby’s bee is a secondary junior homonym of Fabricius’s name. *Lasioglossumparvulum* (Schenck) is now the valid name for Kirby’s bee. More recently, Pauly (1986) described *Homalictusminutus*, which is now transferred to *Lasioglossum* (Danforth and Ji 2001; Gibbs et al. 2012; Ascher and Pickering 2021), making it a secondary junior homonym of *L.minutum* (Fabricius). A new name is required for Pauly’s species, so we propose the replacement name Lasioglossum (Homalictus) minuens. Some authors still maintain usage of *Homalictus* at the generic level (Campbell et al. 2007; Michener 2007; Groom et al. 2013; Niu et al. 2013; Dorey et al. 2019), which would make *L.minuens* a junior synonym of Pauly’s name in that classification based on article 59.4 of the Code.

## ﻿Discussion

Based on the species richness of the relatively well-studied islands of Dominica (26 spp.) to the north and Saint Vincent and the Grenadines (33 spp.) to the south (Ashmead 1900; Moure et al. 2007b; Gibbs 2016, 2020; Ascher and Pickering 2021), it can be expected that the number of bee species on Saint Lucia will eventually rise from ten to at least 25 given sufficient attention. The apid genera *Exomalopsis*, *Melissodes*, and *Xylocopa* and the megachilid genus *Coelioxys* are each known from Dominica, Martinique, and Saint Vincent and the Grenadines (Ashmead 1900; Crawford 1914; Meurgey and Dumbardon-Martial 2015; Meurgey 2016; Ascher and Pickering 2021), making their presence on St. Lucia probable. Furthermore, *Anthophora*, *Melipona*, and *Mesoplia* are known from nearby Dominica and Martinique (Meurgey and Dumbardon-Martial 2015; Meurgey 2016; Ascher and Pickering 2021). Four additional halictid genera, *Augochlora*, *Pseudaugochlora*, *Microsphecodes*, and *Sphecodes* (Ashmead 1900; Crawford 1914; Eickwort and Stage 1972; Gibbs 2016), are also known from the region, so the potential for additional halictid species on St. Lucia is high. Recent studies in the Greater and Lesser Antilles seem to suggest that halictid bee communities are largely distinct between islands (Engel 2001b, 2011; Genaro 2001b, 2021; Gibbs 2018). As such, representatives of these genera could constitute undocumented diversity. Many additional islands in the Lesser Antilles have few or no species of halictid bee known from them. Ongoing work in this area suggests that there are several additional species to describe from smaller islands in the Caribbean. Fourteen morphospecies of Halictidae were recorded from Montserrat, but none with species names (Ivie et al. 2008). In St. Kitts, the only known halictid bee is a brood parasite, but no potential hosts have been documented (Engel 2006b). Additional study of Monserrat, St. Kitts, and other islands in the Lesser and Greater Antilles is needed. This will allow future biogeographical and speciation studies of halictid bees through the Caribbean. Furthermore, baseline data are needed to assess any conservation concerns in the region. As noted previously, several species in the islands show limited distribution within islands and some have not been collected in more than a century (Gibbs 2016, 2018). Targeted surveys for these species would be prudent to determine their status.

## Supplementary Material

XML Treatment for Habralictus

XML Treatment for Habralictus reinae

XML Treatment for Habralictus claviventris

XML Treatment for Dialictus

XML Treatment for Lasioglossum (Dialictus) luciae

XML Treatment for Lasioglossum (Dialictus) cf.dominicense

XML Treatment for Habralictellus

XML Treatment for Lasioglossum (Habralictellus) delphiae

XML Treatment for Lasioglossum (Dialictus) cyaneum

XML Treatment for Lasioglossum (Dialictus) plumbeum

XML Treatment for Lasioglossum (Dialictus) sanctivincenti

XML Treatment for Lasioglossum (Dialictus) gemmeum

XML Treatment for Lasioglossum (Habralictellus) auratum

XML Treatment for Lasioglossum (Dialictus) minutum
